# The Functional Role of Extracellular Matrix Proteins in Cancer

**DOI:** 10.3390/cancers14010238

**Published:** 2022-01-04

**Authors:** Nadezhda V. Popova, Manfred Jücker

**Affiliations:** 1 Laboratory of Receptor Cell Biology, Shemyakin-Ovchinnikov Institute of Bioorganic Chemistry, Russian Academy of Sciences, Miklukho-Maklaya Str., 16/10, 117997 Moscow, Russia; npopova@gmail.com; 2 Institute of Biochemistry and Signal Transduction, University Medical Center Hamburg-Eppendorf, Martinistraße 52, 20246 Hamburg, Germany

**Keywords:** extracellular matrix, tumor microenvironment, tumor progression, matrix metalloproteinases, matrikines, tenascin, fibronectin, collagen

## Abstract

**Simple Summary:**

Extracellular matrix is a three-dimensional network of macromolecules that provide structural and biochemical support to surrounding cells. Extracellular matrix plays a critical role in the development and progression of cancer. The extracellular matrix of the tumor is very different from the matrix of the normal tissue. Mainly fibroblasts produce and regulate matrix remodeling, but in cancer, the tumor matrix also originates from cancer cells. We describe the mechanisms of how the protein composition and structure of the extracellular matrix changes during cancer progression and how abnormal matrix deregulates the behavior of stromal cells and influences cancer progression.

**Abstract:**

The extracellular matrix (ECM) is highly dynamic as it is constantly deposited, remodeled and degraded to maintain tissue homeostasis. ECM is a major structural component of the tumor microenvironment, and cancer development and progression require its extensive reorganization. Cancerized ECM is biochemically different in its composition and is stiffer compared to normal ECM. The abnormal ECM affects cancer progression by directly promoting cell proliferation, survival, migration and differentiation. The restructured extracellular matrix and its degradation fragments (matrikines) also modulate the signaling cascades mediated by the interaction with cell-surface receptors, deregulate the stromal cell behavior and lead to emergence of an oncogenic microenvironment. Here, we summarize the current state of understanding how the composition and structure of ECM changes during cancer progression. We also describe the functional role of key proteins, especially tenascin C and fibronectin, and signaling molecules involved in the formation of the tumor microenvironment, as well as the signaling pathways that they activate in cancer cells.

## 1. Introduction

The tumor microenvironment is a highly heterogeneous environment around a tumor, that includes cellular components (fibroblasts, endothelial cells, adipocytes, immune and inflammatory cells) and a non-cellular component termed the extracellular matrix (ECM). During cancer progression, carcinoma cells recruit host stromal cells, which change their properties and metabolism, and together they create a unique microenvironment to cooperatively remodel the surrounding matrix and promote tumor invasion [1]. Remodeling of the ECM, driven by proteolytic enzymes (such as matrix metalloproteinases) [2], by enzymes that control the modification and cross-linking of extracellular matrix proteins (such as lysyloxidases (LOX)) [3], results in increased stiffness and altered ECM composition. Tumor cells also secrete extracellular vesicles with nucleic acids, lipids and proteins, which can participate in tumor progression and behavior, including tumor environment remodeling, fibroblast activation, angiogenesis, immunomodulation or the establishment of pre-metastatic niches [4,5].

Degradation of ECM is not a passive event as it is accompanied by the release of matrix-bound growth factors as well as matrikines that interact with multiple surface receptors and trigger signal transduction, thus regulating tumor growth and cell migration [6,7].

This review aims to present the functional role of ECM components in tumor development, with particular emphasis on the involvement of fibronectin and tenascin in this process. We also describe the relationships between cancer cells and cancer-associated fibroblasts, and the mechanisms by which cancerized ECM can modulate tumor progression and aggressiveness.

## 2. Structural Organization and Properties of Extracellular Matrix

The extracellular matrix is a major structural component of the tumor microenvironment and is comprised of a three-dimensional network consisting of collagens, laminins, elastin and elastic fibers, glycoproteins and proteoglycans [8]. In normal tissue, ECM provides structural support for the cells and also plays a regulatory role in many cellular processes including growth, migration, differentiation, survival, homeostasis and morphogenesis [9]. Every tissue (e.g., connective tissue, cartilage or bone) has a unique ECM composition, and the components of the ECM are produced and arranged by resident cells in accordance with the needs of the tissue (reviewed in [9]).

**Figure 1:**
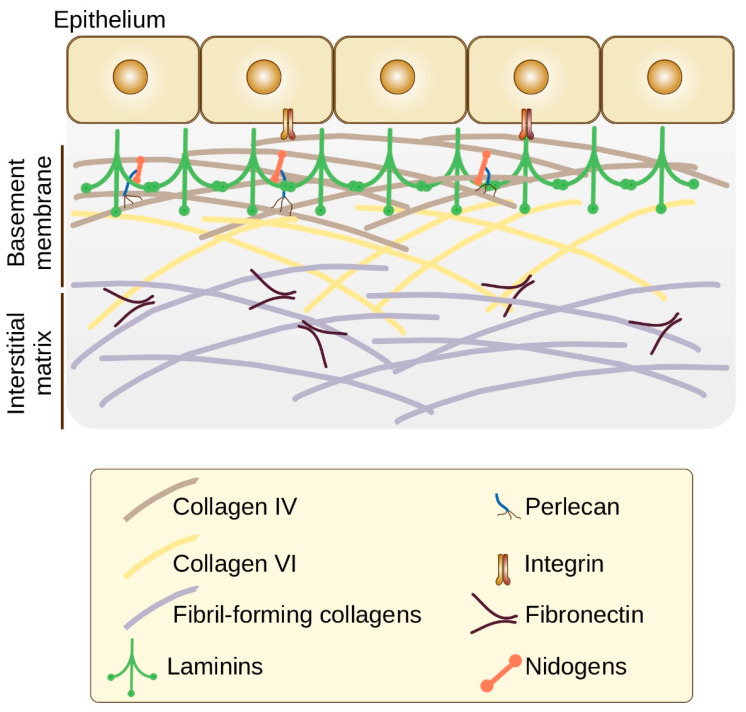
Schematic view of ECM organization (based on [12,17,18]). The major components of the basement membrane are the network-forming collagens (type IV collagen) and laminins. Nidogens and perlecan serve as binding bridges between the two networks: nidogens bridge laminin and collagen IV networks, and perlecan connects nidogen to collagen IV. Collagen VI interact with collagen IV, providing a link between the basement membrane and fibrillar components of the interstitial matrix.

Based on biochemical and structural characteristics, the animal extracellular matrix can be classified into interstitial matrix and basement membrane. The interstitial matrix surround cells, whereas the basement membrane, a thin sheet-like extracellular matrix, delimits the stroma from cells of various origins (epithelial and endothelial cells, neurons and muscle cells, or adipocytes) and surrounds muscle fibers, adipose tissue, Schwann cells as part of myelin nerve fibers [10,11]. The major components of the basement membrane are type IV collagen, laminins, nidogen 1 and 2, and proteoglycans perlecan and agrin (Figure 1) [12].

Collagens are the major proteins of the ECM. Collagens are typically homo- or heterotrimers made of one, two or three different polypeptide chains (α-chains) [13]. For example, there are six different genes designated *COL4A1-COL4A6* encoding type IV collagen chains α1(IV)–α6(IV) [14]. Laminins are large heterotrimeric glycoproteins, consisting of α, β and γ chains that assemble to form a Y-shaped molecule. Mammalian genomes encode five α (α1–α5), four β (β1–β4) and three γ (γ1–γ3) chains [15] and the laminin isoforms are named according to the three chain composition: i.e., laminin 332 is composed of α3, β3 and γ2 chains [16].

**Figure 2:**
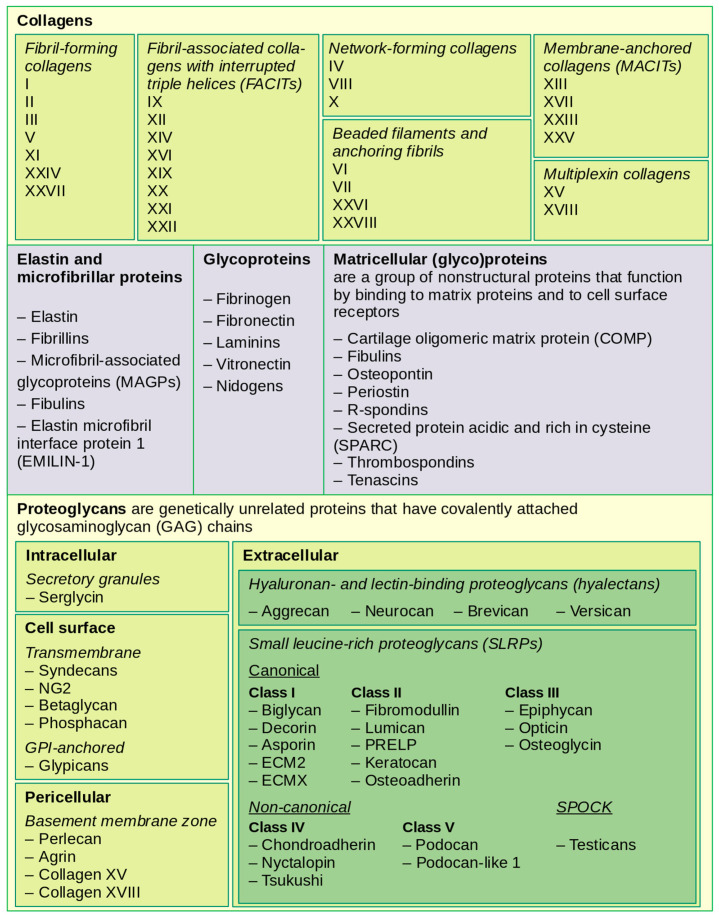
Structural-functional classification of matrix proteins (based on [13,18,24,25,26]).

Type IV collagen and laminin individually self-assemble into two independent but interconnected networks that serve as the foundation for basement membrane [18,19,20]. Globular domain of laminins at the end of the α chain binds to cellular receptors, including integrins, α-dystroglycan, heparan sulfates and sulfated glycolipids [17,21]. Nidogens and proteoglycans perlecan and agrin bridge the laminin and type IV collagen networks, increase their stability and influence the structural integrity of basement membrane [22]. Basement membrane also contains matricellular proteins such as thrombospondins, secreted protein acidic and rich in cysteine (SPARC), cartilage oligomeric matrix protein (COMP), tenascins and osteopontin (Figure 2) that interact with other matrix components and growth factors as well as with cell surface receptors contributing to tissue-specific functions [23,24].

Interactions of cells with the ECM are mediated by their surface receptors, such as integrins, syndecans and discoidin domain receptors (DDRs) [8]. The family of integrins that are the main cell adhesion receptors for components of ECM includes at least 24 transmembrane heterodimers generated from a combination of non-covalently paired α- and β-subunits. Based on ligand substrate, integrins can be classified into receptors recognizing Arg-Gly-Asp (RGD) peptide motifs, collagen, laminin or leukocyte-specific integrins [27,28]. Integrins are bi-directional signaling receptors involved in outside-in and inside-out signaling. The inside-out signaling mainly acts to bring the integrin into the active conformation [29]. Upon ligand binding, integrins undergo conformation changes leading to outside-in signaling. Ligand-bound integrins engage the actin network via talin and additional cytoskeletal linker proteins, leading to integrin clustering and the following activation of focal adhesion kinase (FAK), Src-family protein tyrosine kinases and integrin-linked kinase (ILK) [28,30]. Integrin adhesion also activates the Ras-ERK, PI3K/AKT and YAP/TAZ pathways [31,32].

Basement membranes are tightly associated with the interstitial matrix (lamina propria) through collagen fibrils, including collagens VI and VII. Collagen VI binds to laminin and collagen IV, as well as to collagen I fibrils in the interstitial matrix [33,34,35]. The protein composition of the interstitial matrix mainly includes collagens (I, III, V, VI), fibronectin and elastin [18].

## 3. Fibroblasts and Cancer-Associated Fibroblasts

ECM is a highly dynamic structure that is constantly remodeled, and fibroblasts are the major producers of ECM in normal physiology and in tissue repair [36]. After injury, tissue healing begins by activation of platelets to form a clot consisting of fibrin and fibronectin [37,38]. Platelets recruit immune cells to the injury site by releasing abundant soluble mediators and also produce adhesive glycoproteins resulting in the deposition of a fibrin clot that serves as a provisional matrix [39]. Platelets, endothelial cells and macrophages secrete signaling molecules including transforming growth factor (TGF-β) and platelet-derived growth factor (PDGF) that recruit resident and immigrating fibroblasts. These signals direct fibroblasts to either obtain pro-fibrotic phenotype, switching to ECM protein synthesis, or differentiate into myofibroblasts which participate in wound contraction [40]. Fibroblasts degrade the provisional matrix [41] by producing matrix metalloproteinases (MMPs) and replace it with immature collagens, elastin, fibronectin and proteoglycans, and forming mature collagen fibrils later in repair [39]. As the wound closes and evolves into a scar, myofibroblasts become apoptotic and finally disappear [42].

TGF-β, among other inflammatory cytokines, is a key regulator of fibroblast to myofibroblast differentiation during wound healing and cancer-associated fibroblasts (CAFs) transition in various cancers [43], including prostate, breast, pancreatic, bladder and colorectal cancer [44,45,46,47,48,49,50]. Active TGF-β can be released from ECM as a result of its degradation (reviewed in [51]) or can be secreted in exosomes by cancer cells [47]. CAFs in turn can also derive not only from resident fibroblasts and myofibroblast-like cells [52] but also from adipocytes [53], bone marrow-derived mesenchymal cells [54,55], hematopoietic stem cells [56,57] or endothelial cells [58], which explains, at least in part, the heterogeneity of CAFs in the tumor microenvironment [59,60].

Since CAFs produce a number of proteins that are specific to the origin of the cells and there is no specific protein to CAFs, a combination of proteins is used as markers to identify CAFs. Several intracellular and plasma membrane-associated proteins such as alpha-smooth muscle actin (α-SMA), fibroblast-activating protein (FAP), S100A4 protein/fibroblast specific protein-1 (FSP1) and vimentin have been used as CAF markers [60,61,62,63,64]. CAFs are characterized by increased proliferative capacity, elevated production of growth factors and ECM proteins, and increased metabolic activity [65,66,67]. Cancer-associated fibroblasts have been found to produce highly aligned fibronectin matrix, which promotes directional migration of cancer cells in CAF-derived matrices [68,69].

## 4. ECM Remodeling and Modification in Cancer

ECM deposition is considered a hallmark of cancer [70]. Mainly CAFs are responsible for ECM synthesis and remodeling. CAFs, driven by cancer cells, not only synthesize, produce and deposit substantial amounts of ECM components [71] but also contract the tissue, thus altering the ECM of the tumor stroma qualitatively and quantitatively [72]. Some tumor cells themselves can also synthesize components of the ECM such as collagen [73,74] and other ECM- and ECM-associated proteins [75,76], including secreted factors and modulators of the matrix [77]. Compared to stromal cells, cancer cells produce less amount of ECM proteins (<10% in pancreatic cancer [78]) but it was found that a number of cancer-cell–derived proteins can promote tumorigenesis and metastasis and correlate with poor patient survival [78,79].

### 4.1. Collagen Reorganization

Increased deposition of collagen is the most common alteration of ECM in cancer. In healthy tissues, interstitial collagen fibers are wavy, whereas the collagen fibers in tumors are often straightened [80,81,82] as well as quantitatively and qualitatively reorganized [83,84]. 

Three different orientations and deposition of collagen fibers, named tumor-associated collagen signatures (TACS), were revealed in human breast cancer to characterize tumors: TACS-1, the increased collagen fiber accumulation at a region surrounding the small tumors; TACS-2, the presence of straightened collagen fibers stretched around the tumor in noninvasive tumors and lastly TACS-3, the presence of collagen fibers aligned perpendicular to the tumor boundary [82]. Similar structural collagen signatures were found in other cancers such as ovarian cancer [85,86], basal cell carcinoma [87,88], prostate cancer [88], glioblastoma [89], renal cell carcinoma [80] and pancreatic ductal adenocarcinoma [90,91]. TACS have been shown to promote focal invasion and metastasis [82], influence disease state and tumor invasion [92,93] and correlate with significantly worse patient survival in multiple tumor types [81,84,90,94,95]. 

Linearly aligned collagen fibers are thought to generate migration highways that allow cancer cell invasion and dissemination [96]. It was demonstrated that ECM deposited by CAFs was remarkably aligned in a parallel pattern, and CAFs promoted the dissemination of malignant cells in vivo [97]. Increased collagen density and enzymatic cross-linking of collagen during tumor progression can lead to matrix stiffening, and stiffened cross-linked fibrillar collagen promoted enhanced PI3 kinase (PI3K) activity, and induced the invasion of an oncogene-initiated epithelium [98].

To locally degrade and to penetrate ECM barriers, cells use actin-rich protrusions termed invadosomes, which include invadopodia (in cancer cells) or podosomes (in stromal cells) [99]. It was shown that a high-density fibrillar collagen matrix can itself induce a formation of invadopodia via a specific integrin signaling pathway even in the absence of a significantly altered gene or specific protein expression [100]. Collagen I and IV have been demonstrated to promote invadopodia extension and migration of tumor cells as well as fibroblasts, endothelial cells and macrophages [101,102].

Some collagens also bind and activate receptor tyrosine kinases discoidin domain receptors (DDR1 and DDR2) that are thought to mediate metastases and cancer aggressiveness [103,104,105,106]. DDR1 activation induces Src, Notch and IKK signaling pathway, DDR2 promotes oncogenic signaling via mTORC2 and AKT pathway, and recent findings have demonstrated that DDR-collagen signaling plays an important role in cancer progression and metastasis (reviewed in [107,108,109]).

### 4.2. Collagens and Laminins

Many types of collagen are upregulated in cancer and involved in almost all steps of tumor progression including proliferation, invasion, angiogenesis and metastasis [110,111]. 

Fibril-forming collagens provide three-dimensional frameworks of tissues and organs, and their involvement in cancer progression has been the most studied in comparison to other types of collagens [112]. The most abundant type I collagen plays a significant role in different cancers and metastases [113,114,115,116,117]. Type I collagen-dense ECM can drive the metastases of estrogen receptor 1 (ERα+) breast cancers by altering hormonal signals [118]. It was also demonstrated that type I collagen can induce resistance to epidermal growth factor receptor tyrosine kinase inhibitors (EGFR-TKIs) via mTOR activation through an AKT-independent pathway [119,120]. Type XI collagen, a minor fibril-forming collagen, is a component of fibrils both in cartilage and a wide variety of non-cartilaginous tissues [121,122]. Its alpha 1 chain (COL11A1) has been found to be upregulated in a variety of cancers [123,124,125,126,127,128] and is supposed to promote proliferation, angiogenesis, invasion and drug resistance of cancer cells [129,130,131,132]. Proteolytic products of type XI collagen released into the circulation can be used as predictive markers for patients with pancreatic ductal adenocarcinoma [133]. Type V collagen, another minor fibril-forming collagen, was also shown to be upregulated in squamous cell carcinoma, colorectal, gastric and breast cancer and associated with poor prognosis [134,135,136,137].

Collagen VI is expressed in many tissues where it interacts with different ECM proteins and cell surface molecules and functions as a bridge between the basement membrane and interstitial matrix [138,139]. Type VI collagen expression is elevated in many human cancers such as pancreatic, ovarian, lung, breast and colon cancer, favoring cell survival and tumor progression [140,141,142,143,144,145]. In recent studies, it has been demonstrated that alpha-3 chain of collagen VI and the cleaved C5 domain fragment, called endotrophin [146], are highly expressed in a variety of cancers and play a crucial role in tumorigenesis [147,148].

Laminins are also involved in cancer progression, including invasion, migration, angiogenesis, metastasis and drug resistance [149,150,151]. Expression of various laminin subunits is often upregulated in tumor or stromal cells of malignant tissues. Laminin-332 has attracted a lot of attention by its altered expression pattern in several human carcinomas: expression of laminin-332, or its γ2 chain, is markedly elevated in mammary, colon, melanoma and sarcoma cancer cells and correlates with tumor invasiveness and poor patient prognosis [152]. It is proposed that laminin-332 involvement in tumor progression is mediated by binding to integrins α6β4 or α3β1 [153,154,155,156] and subsequent activation of focal adhesion kinase (FAK) and extracellular signal-regulated kinase (ERK) [156,157]. Increased laminin-332 γ2 chain expression is a significant poor prognostic indicator for the patients with esophageal or colorectal carcinoma [158,159]. The fragment of laminin-332 γ2 chain after cleavage by MT1-MMP [160,161] or MMP-2 [162] can bind to and activate EGFR and downstream MAPK signaling in cancer cells as well as MMP-2 gene expression and cell migration [163].

### 4.3. Fibronectin

Fibronectin is a high-molecular-weight glycoprotein that consists of two subunits, covalently linked by a pair of disulfide bonds at the C-termini [164,165]. Despite fibronectin protein being produced from a single gene, there are 20 isoforms of human fibronectin as a result of alternative splicing [166,167,168]. Fibronectin exists soluble as a dimer in the plasma (pFN) or as an insoluble part of the ECM (cellular FN) [169] where it interacts with many other ECM components. Fibronectins have been shown to interact with integrins, collagen, tenascin-C, fibrillin, glycosaminoglycans as well as with growth factors (reviewed in [170]).

**Figure 3:**
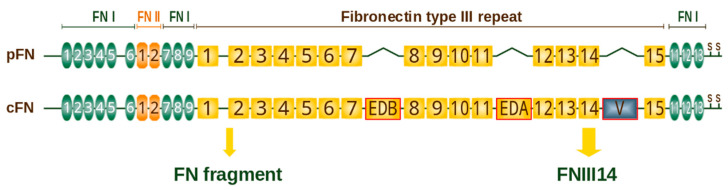
Domain structure of human fibronectin (based on SMART-tool [176] and review [177]). Each monomer contains three types of repeats: fibronectin type I (green ovals), type II (orange) and type III (gold rectangles). One or both of the FN type III modules (ED-A or ED-B) may be present in cellular fibronectin (cFN) but never in plasma fibronectin (pFN). A “variable” V or IIICS region is located between FNIII14 and FNIII15 and spliced out in ~50% subunits of pFN [178]. Two subunits are linked by a pair of C-terminal disulfide bonds to form a protein dimer. FNIII14 and “FN fragment” are proteolytic bioactive fragments derived from fibronectin.

Plasma fibronectin is synthesized and secreted without ED-A or ED-B segments (Figure 3) by hepatocytes [171]. Cellular fibronectin is synthesized by many cell types, including fibroblasts, endothelial cells, macrophages and tumor cells, and secreted with ED-A and/or ED-B extra-domains [172,173]. In cancer, CAFs have generally considered a source of fibronectin [174,175]. CAFs secrete and assemble fibronectin as parallel fibers mediating directional cancer cell migration [68,69].

For instance, in breast tumor tissues the expression of fibronectin, and specifically the ED-B isoform, was significantly higher compared to the adjacent tissues. Immunohistochemical analysis showed ED-B expression in cancer cell-associated fibroblasts, stroma and stromal fibroblasts, as well as in primary breast tumor and lymph node, lung, and brains metastases [179]. The expression of fibronectin is elevated in many solid tumors and is supposed to correlate with tumor grade/aggressiveness and can serve as a prognostic marker [174,180,181,182,183,184].

Although it is assumed that the role of fibronectin in oncogenesis and malignant progression is highly controversial [185] the mechanisms of fibronectin action in cancer are being actively studied (reviewed in [186,187,188]). 

The role of fibronectin in promoting growth, survival and invasion of cancer cells has been highlighted by in vitro studies. It was shown that detachment of pancreatic cancer cells from ECM stimulated necrosis but fibronectin and laminin markedly increased the cells’ survival by inhibiting both mitochondrial dysfunction and caspase activity [189]. Then this group of authors provided evidence that in pancreatic cancer cells fibronectin increased intracellular reactive oxygen species (ROS) production and NADPH oxidase activation [190]. The prosurvival effect of fibronectin on pancreatic cancer cells has been further investigated and shown to be mediated through the trans-activation of IGF-IR. The mechanism involved fibronectin-mediated complex formation between integrin β3 and protein-tyrosine phosphatase SHP-2 that prevented SHP-2 from dephosphorylation of IGF-IR resulting in its sustained phosphorylation and the downstream activation of AKT kinase, up-regulation of anti-apoptotic Bcl-xL and inhibition of apoptosis [191].

It was also found that exogenous fibronectin stimulates lung carcinoma cell proliferation via integrin α5β1 through activation of the AKT/mTOR/p70S6K pathway and inhibition of AMPK and LKB1 expression [192]. Exogenous fibronectin significantly enhanced proliferation and invasion in gallbladder cancer cell lines and markedly activated AKT/mTOR/4E-BP1 signaling cascade [193]. The role of fibronectin in promoting growth and migration via Src and TGF-β1 signaling was also demonstrated in renal cell carcinoma [194]. Fibronectin can support cancer cell proliferation through Erk and Rho-kinase signaling [195]. A recent study provided evidence that collagen and fibronectin together, but not alone, facilitate proliferation and tumorigenesis of glioma cells through PI3K/AKT/SOX2 and CDC42/F-actin/YAP-1/Nupr1/Nestin signaling pathways via integrin αvβ3 [196]. 

Fibronectin is also a cargo in extracellular vesicles (EV) from different cell types (reviewed in [197]). EV is a term used to define lipid bilayer membrane particles secreted by cells [198]. EVs can be generally divided into exosomes, microvesicles, apoptotic bodies and recently identified matrix-bound nanovesicles (MBV) [198,199,200]. Exosomes have been shown to play a major role in different stages of cancer progression, including cell proliferation, angiogenesis, invasion and metastasis [201,202,203]. MBVs, vesicles integrated within the dense fibrillar network of the ECM [200], are differ from exosomes in membrane composition and luminal cargo [204]. MBVs are supposed to have a potential role in development, homeostasis, wound healing, tissue regeneration and neoplasia [205], but to date, there is no information on their involvement in cancer.

Fibronectin was found on the surface of fibroblast-derived extracellular vesicles. The EV surface–associated fibronectin induced an invasive phenotype in recipient fibroblasts, and the effect was dependent on interaction with the fibronectin receptor α5β1 integrin, and activation of FAK and Src family kinases [206].

In a mouse model, circulating fibronectin enhanced the amount of local fibronectin in tumors through a positive feedback loop as well as enhanced tumor growth by increasing vascular endothelial growth factor (VEGF) content and VEGF-mediated signaling [207].

The production of fibronectin by cancer cells also contributes to the tumor development. In suspension cultures, squamous cell carcinoma cell aggregates, but not single cells, had high levels of fibronectin and were more resistant to anoikis through a mechanism involving fibronectin and the integrin αv receptor/FAK signaling [208]. An upregulation of fibronectin levels due to detachment has also been demonstrated for lung and breast cancer cell aggregates, and this upregulation was suggested to be important for the development of anoikis resistance and further metastasis [209]. In a mouse model, a decrease in fibronectin production by cancer cells was shown to inhibit cancer growth due to inhibition of proliferation by decreasing ERK phosphorylation and diminishing YAP expression [210].

### 4.4. Tenascin

Tenascin-C, the first discovered member of the tenascin family, is a multifunctional ECM glycoprotein [211,212,213,214]. Other members of the family are tenascin-R, tenascin-W, tenascin-X and tenascin-Y [215].

**Figure 4:**
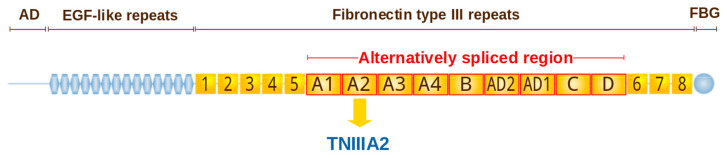
Domain structure of human tenascin-C (based on SMART-tool [176] and review [226]). N-terminal assembly domain (AD) links the tenascin chains and mediates the oligomerization and formation of hexamers. The assembly domain is followed by the EGF-like repeats and two types of FN-III domains: conserved (yellow rectangles) and alternatively spliced (yellow with red), and the C-terminal fibrinogen-like domain (FBG). Tenascin-C-derived 22-mer peptide TNIIIA2.

The molecule of tenascin-C consists of six ~250 kDa subunits that are linked together at one end by disulfide bonds, and each subunit has four distinct domains [216,217,218]. Eight FNIII repeats (FNIII 1–8) are constitutively present in the tenascin-C molecule (Figure 4), but the nine FNIII repeats located between the fifth and sixth FNIII domain may be present due to alternative splicing [219]. The alternative splicing is thought to control the tenascin-C versatile functions by modulating its interaction with specific binding partners [219]. Moreover, splice isoforms demonstrate different sensitivity to proteases [220]. Tenascin-C has been shown to interact with various ligands, for instance with integrins [221], EGFR [222], fibronectin [223,224] and other ECM components (reviewed in [225]).

Tenascin-C has highly specific and restricted expression patterns in the embryo [227] but is almost undetectable in most adult tissues [228]. In adults, tenascin-C expression can be upregulated by mechanical stress, upon tissue repair or in cancer (reviewed in [228,229]). By mechanical stress, tenascin-C expression was found to be regulated by dynamic (cyclic) strain [230,231] as well as by static tensile stress, for instance by stressed collagen gels [232]. Tenascin-C expression is elevated in fibroblasts in response to TGF-β [233,234] or PDGF via PI3K/AKT pathway [235] upon inflammation or wound repair (reviewed in [229]). Although the expression of tenascin-C in the adult tissue is usually low, it has been described to be elevated in many human cancers [236,237]. 

Tenascin-C is highly expressed by cancer-associated fibroblasts and stromal cells, as well as by some cancer cells, and has been involved in promoting proliferation, migration, angiogenesis and metastasis [237,238,239,240,241,242,243,244]. High expression of tenascin-C correlates with cancer grade and poor prognosis in various cancers [245], including esophageal squamous cell carcinoma [246], glioma [247], gastric [248], prostate [249], breast [250] and colorectal cancer [251,252,253]. 

Expression of tenascin-C in tumor stroma was found in regions called tumor matrix tracks that are composed of several matrix molecules and are enriched with stromal cells including endothelial cells, fibroblasts and immune cells [243,254,255]. It was also found that tenascin-C and thrombospondin-2 co-localize with aligned collagen fibers in patient samples with invasive ductal carcinoma [256]. Tenascin-C expression can be induced in cancer cells as well as in stromal cells by different factors, including EGF, TGF-β, b-FGF and TNF-α [219,229]. For instance, mammary tumor cells produce TGF-β1, which induces fibroblasts to synthesize tenascin [257]. Ras-transformed epithelial cells express tenascin-C in response to TGF-β, and elevated ERK/MAPK signaling but not PI3K signaling was required for high expression levels of tenascin-C [258].

In vitro experiments suggested that tenascin-C can induce epithelial-to-mesenchymal transition (EMT) in breast cancer cells via binding to αvβ6 and αvβ1 integrins [259], colorectal [260] and pancreatic cancer cells [261] or promote invadopodia formation in Ewing sarcoma [262]. Knockdown of tenascin-C inhibited the proliferation and invasion of gastric cancer cells and EMT process through inhibited ERK phosphorylation [248]. Tenascin-C has been found to induce and activate others signaling pathways in cancer cells such as JNK, Wnt, Notch, AKT/HIF1α and TGF-β [241,242,244,261,263,264,265]. 

The effect of tenascin on stromal formation has also been investigated. The addition of tenascin-C to fibroblasts significantly up-regulated α-smooth muscle actin and calponin that are the specific markers of differentiation to myofibroblasts, as well as significantly up-regulated the production of tenascin itself through integrin αvβ1/TGF-β signaling axis [266]. Yeo et al. demonstrated that tenascin-C is not only produced from fibroblasts but also is essential for their activation. They have found a positive feedback loop comprising basic helix-loop-helix transcription factor Twist1, homeobox transcription factor Prrx1, and tenascin-C functions as a bistable (ON/OFF) switch to initiate fibroblast activation [267].

Like fibronectin, tenascin-C was also detected in extracellular vesicles released into the extracellular space by most cell types, including fibroblasts and tumor cells (reviewed in [197]). Moreover, tenascin-C was detected in exosomes isolated from the blood of glioblastoma patients [268]. The authors established an immunosuppressive role for tenascin-C, since it was shown that tenascin-C inhibited T-cell proliferation through interaction with integrins α5β1 and αvβ6 on T lymphocytes and decrease in AKT/mTOR signaling [268]. Jachetti et al. have also demonstrated that tenascin-C produced by prostate cancer stem-like cells inhibited T-cell proliferation by interacting with α5β1 integrin and blocking reorganization of the actin-based cytoskeleton [269].

Experiments in vivo showed that knockdown of tenascin-C inhibited tumor growth and peritoneal dissemination and suggested the involvement of tenascin-C in vasculogenic mimicry formation [248]. It was also shown that stromal-derived tenascin-C promotes lung metastasis by impacting blood vessels invasions (BVI) at multiple levels [244]. Tenascin-C may generate barriers for infiltrating T lymphocytes (TIL), thus contributing to the escape from anti-tumor immunity [270].

In cancer, not only increased expression of tenascin is observed, but also various splice isoforms generated through alternative splicing of exons within fibronectin type III repeats (reviewed in [219,271]). Certain splice isoforms of tenascin-C were found in particular types of cancer [236]. Some studies have shown that larger tenascin-C isoforms are expressed only in tumorous but not in healthy tissues and their expression correlate with cancer progression and poor prognosis in glioma [272], bladder [273,274], ovarian [275] breast [276,277], pancreatic [278], esophageal [246] and lung cancer [279]. However, there are also data that the higher molecular weight of the isoform may not be the most relevant to cancer progression as there are examples of cancers where the smallest tenascin-C isoform is predominantly expressed or smaller splice variants promote invasion more effectively than does the unspliced tenascin-C [280,281,282].

## 5. Degradation of the Tumourigenic Matrix

### 5.1. Matrix Remodeling Enzymes

To permit invasion and cell migration, the extracellular matrix must be destroyed. The ECM is cleaved and degraded by target-specific proteases such as matrix metalloproteinases (MMPs), a disintegrin and metalloproteinases (ADAMs), a disintegrin and metalloproteinases with thrombospondin motifs (ADAMTS), cathepsins and plasminogen activation system components (reviewed in [2,283,284]). These proteases are secreted primarily by stromal cells but also by cancer cells [285]. It was found in several studies in vitro and in vivo that CAFs induce migration, invasion and metastasis of cancer cells by producing various MMPs, including MMP-1, -2, -3, -7, -9 and -14 [286,287,288,289,290].

Matrix metalloproteinases (MMPs) are a large family of calcium-dependent zinc-containing endopeptidases: 28 different MMPs were found in vertebrates, of which at least 23 MMPs are expressed in humans [291,292]. On the basis of their substrate specificity and domain organization, MMPs are classified into collagenases, gelatinases, stromelysins, matrilysins, membrane-type (MT)-MMPs and other MMPs [292]. MMPs are initially secreted as inactive zymogens [293] due to the interaction of a cysteine residue of the pro-domain with the zinc ion of the catalytic site. Only after disruption of this interaction by proteolytic removal of the pro-domain the enzyme become proteolytically active [294,295,296].

Gelatinases MMP-2 and MMP-9 as well as transmembrane MMP-14 (also known as MT1-MMP) are enriched at invadopodia that are essential for the degradation of ECM components and cell invasion [297,298,299,300]. A number of signals from the tumor environment, such as growth factors, hypoxia, extracellular pH, metabolism or direct interactions with stromal cells affect invadopodia formation and function [301,302]. The number and activity of invadopodia can also be directly increased by ECM rigidity, indicating a potential mechanism for the reported correlation of tissue density with cancer aggressiveness [303,304].

MT1-MMP (MMP14) is considered to play a key role in the early stages of tumor invasion and cancer progression [305]. Cancer cells concentrate MT1-MMP at invadopodia membranes [306,307,308,309] that can degrade a number of ECM molecules including collagen types I, II, III, fibronectin, tenascin, nidogen, perlecan and aggrecan [310,311]. MT1-MMP also activates MMP-2 [312,313] and combined action of MT1-MMP and MMP-2 is thought to enhance ECM proteolysis: MT1-MMP denaturates collagen into gelatin, which is subsequently digested by MMP-2 gelatinase [314]. Elevated expression of MMP-2 and MMP-9 is indeed considered a hallmark of cancer aggressiveness, and it is suggested that their levels can be useful prognostic biomarkers [315,316]. As a result of basement membrane degradation, tumor cells gain access to the blood and lymphatic vessels. As a result of excessive collagen remodeling by matrix metalloproteinases, small protein fragments of degraded collagens are released into the circulation and can be used to measure tumor activity and invasiveness as well as to serve as a prognostic and/or predictive biomarkers in different cancers [317,318,319].

### 5.2. Matrikines

Proteases secreted into the tumor microenvironment are not only essential for ECM remodeling, but they also release bioactive ECM fragments matrikines [320,321] or expose matricryptins [322]. These fragments in turn can regulate a wide array of biological processes, including angiogenesis, cell migration, adhesion and differentiation as well as tumor growth and metastasis [323]. It was found that matrikines can originate from collagen [324,325], elastin [326,327], tenascin [328,329], fibronectin [330,331], laminins [332], decorin, thrombospondin and versican [333].

In cancer, the most well-studied matricryptins are derived from collagens (e.g., collagens I, IIB, IV, VIII, XV, XVIII and XIX) [334]. A number of well-studied collagen fragments have anti-tumorigenic properties: arresten [335], canstatin [336,337,338], endostatin [339,340,341], pentastatin [342] and vastatin [343,344]. In contrast, there are matrikines that exhibit pro-tumorigenic activity. For instance, endotrophin plays a critical role in cancer development and is considered to be a target for anti-tumor therapy [139,148,345].

Tenascin-C is also proteolytically cleaved by MMP-2 and cathepsin B [346,347], and degradation was associated with higher recurrence and a worse prognosis for the patients with lung cancer [346,348]. It was found that tenascin-C molecule contains cryptic sequence YTITIRGV within the FN type III repeat A2 (Figure 4) that is exposed by MMP-2 processing [349]. The 22-mer peptide containing YTITIRGV termed TNIIIA2 is capable to induce activation of integrin α5β1 and to rescue non-transformed fibroblasts from serum starvation–elicited apoptosis through activation of the AKT/Bcl-2 pathway [350]. It was also found that activation of integrin α5β1 by TNIIIA2 caused active proliferation, disseminative migration and anoikis resistance in glioblastoma cells [351,352]. In glioblastoma cells, TNIIIA2 was also able to stimulate PDGF production that resulted in upregulation of the tenascin-C expression in these cells. Tenascin-C induced increased expression of the active form of MMP-2 in glioblastoma cells, which was supposed to contribute to continuous PDGF production suggesting a positive feedback loop of tenascin-C/TNIIIA2/PDGF leading to hyper-proliferation of glioblastoma cells [353,354]. TNIIIA2 also promoted colon cancer cell invasion in vitro by upregulating MMPs and boosted pulmonary metastasis of colon cancer cells in a mouse model [355].

Unlike TNIIIA2, which has the ability to activate β1-integrins and is involved in the aggressive development of cancer, FNIII14, the peptide containing the bioactive site of plasma fibronectin, demonstrated an anti-tumor effect [329]. It was found that plasma fibronectin has a cryptic functional site YTIYVIAL, termed FNIII14 within the 14th FN type III repeat (Figure 3) [356]. FNIII14 was shown to attenuate the TNIIIA2-induced aggressive phenotype of glioblastoma cells through β1-integrin inactivation as well as delayed glioblastoma growth in a mouse xenograft model [352]. Another proteolytic fragment of fibronectin (Figure 3) secreted by bone marrow-resident mesenchymal stromal cells induced chemotaxis of prostate cancer cells via classic fibronectin-binding integrins α5β1 [330]. It was supposed that proteolytic fragments of fibronectin may function as matrikines in the bone marrow niche and are involved in seed-and-soil mechanisms of disease progression [330]. 

Elastin is highly resistant to proteolysis and shows essentially no turnover in healthy tissues [357]. However, MMPs, aspartic proteases, serine proteases and cysteine proteases can fragment elastin to elastin peptides under several pathological processes, and elastin fragments can, in turn, activate MMPs (reviewed in [358]). In melanoma, fragmentation of elastin was found to occur at the invasive front of the tumor. Elastin fragments enhanced MMP-2 and MMP-14 production by melanoma cells that allowed further melanoma cell invasion through a type I collagen matrix by upregulating MMP-2 expression and activation [359]. 

Two categories of elastin-derived peptides have been described: Val-Gly-Val-Ala-Pro-Gly (VGVAPG, VG-6) with a xGxxPG consensus and Ala-Gly-Val-Pro-Gly-Leu-Gly-Val-Gly (AGVPGLGVG, AG-9) with the xGxPGxGxG consensus sequence [360,361]. Elastin peptide VGVAPG has been shown to stimulate fibrosarcoma cell invasion through the activation of MMP-2 and uPA [362,363], upregulate MMP-14 and stimulate the angiogenic phenotype of endothelial cells [364] and increase the invasiveness of lung cancer cells by post-transcriptional regulation of MMP-2 and uPA [365]. VGVAPG increased the migration of melanoma cells and the generation of elastin-derived peptides, enhancing the expression of elastin-degrading MMP-2 and MMP-3 through binding to galectin-3 and EBP receptors. VGVAPG also increased cells attachment and the expression of major adhesion molecules CD44, ICAM-1 and NCAM on melanoma cells through galectin-3 and integrin αvβ3 receptors [366]. The other peptide, AG-9, has been also shown to induce tumor growth, MMP-2 and uPA secretion, cell migration and cell adhesion [361]. The pro-invasive effects of AG-9 in squamous carcinoma cells were mediated through the ribosomal protein SA (RPSA) receptor [367].

## 6. Future Perspectives and Approaches in ECM Research

As outlined above, there are numerous publications implicating ECM proteins in cancer progression. We would like to outline here the main directions and methodological approaches for studying the role of ECM proteins in cancer.

### 6.1. Animal Models

In future experiments, experimental mouse models have to be established to further prove the functional role of ECM proteins in the process of tumor metastasis. There have been already several knockdown experiments of ECM proteins performed and analyzed in xenotransplantation mouse models. Recently, knockdown of tenascin-C in a human gastric cancer cell line has been shown to be implicated in the process of epithelial-mesenchymal transition and inhibited subcutaneous tumor growth and peritoneal metastasis in a xenotransplantation mouse model in vivo [248]. In further experiments, tissue-specific knockouts by using e.g., the Cre/LoxP system have to be made in order to analyze the tissue-specific function of ECM proteins during the process of metastasis in distinct organs [368]. Alternatively, by using the same Cre/LoxP system, tissue-specific overexpression of ECM proteins can be induced in order to analyze their functional role in metastasis.

An additional aspect is the functional role of the tumor microenvironment in mediating the functional role of ECM proteins. As outlined above, distinct signaling processes have been implicated in the functional role of ECM proteins in mediating the metastatic process. Therefore, distinct conditional and/or organ-specific knockout mice with deletions in the proposed signaling molecules like EGF receptors, WNT signaling, RAS-RAF-MAPK and PI3K-AKT-mTOR signal transduction have to be used to further pin down these signaling events in the tumor-infiltrating stroma cells after metastasis in distinct organs.

As we have already mentioned, there is a close interplay between tumor cells and the surrounding microenvironment. Tumors, arising from a single cell, gradually develop into heterogeneous cancer cell populations. Thus, mouse models can also be used to study tumor heterogeneity [369]. By using red, green, blue (RGB) marking as a lentiviral multi-color clonal cell tracking technology, the clonal development of subpopulations within tumors and metastases can be analyzed after transplantation of the RGB marked cells in mice [370]. The combination of RGB marking with immunohistochemical staining of distinct ECM proteins with fluorescently labeled antibodies will give additional information of the functional role of ECM proteins during the process of clonal development in tumorigenesis and/or metastasis.

### 6.2. Proteomic Approach

By analyzing the structure and functions of ECM, the proteomics-based and bioinformatic methods are undoubtedly needed to be mentioned. Proteomic characterization of matrisomes (“a list of all the proteins in any given matrix”) [371] or integrin adhesomes can be performed using mass spectrometry methods [76,372,373].

### 6.3. Imaging Technologies

ECM, and especially cancerized ECM, can be characterized not only by the protein composition and spatial organization but also by such a parameter as stiffness. As it was mentioned in the review, ECM remodeling, driven by proteolytic enzymes and cross-linking enzymes results in increased stiffness of ECM surrounding the tumor [374]. Sometimes conventional cell and molecular biology methods are not enough to characterize complex physico-chemical properties of ECM in cancer.

High-resolution microscopy techniques, for instance, second harmonic generation (SHG) or coherent anti-Stokes Raman scattering (CARS), are successfully used to quantify tissue structural changes during cancer progression [375,376]. It was also reported about the combined use of SHG microscopy and mass spectrometry [377].

### 6.4. 3D Cell Models and Tissue Engineering

Engineering approaches can be used to examine the effects of tumor-associated alterations in the ECM or ECM composition [378]. To explore the molecular mechanisms of tumor progression and metastasis, 3D cancer models can be used for the imitation of key steps of cancer dissemination (invasion, intravasation and angiogenesis) [379,380]. The decellularized matrix allows a comprehensive study of the ECM role in the regulation of cancer cell behavior [381,382].

## 7. Conclusions

In this review we have presented a picture of the versatile role of ECM proteins in cancer progression. Researchers focus now not only on the intrinsic properties of cancer cells and consider cancer not only as a disease of uncontrolled cell proliferation, but as a complex interplay of cancer and stromal cells in the dysregulated microenvironment.

To understand the mechanisms of ECM deregulation in cancer, it is crucial to elucidate the molecular basis of the cancer-dependent ECM remodeling, changes in gene expression and ECM composition, and the involvement of certain signaling pathways. Understanding the molecular mechanisms underlying survival, proliferation and invasion will help in the development of new therapeutic agents targeting tumor development and metastasis.

## Abbreviations

ADAM	disintegrin and metalloproteinases
ADAMTS	a disintegrin and metalloproteinases with thrombospondin motifs AMPK AMP-activated protein kinase
b-FGF	basic fibroblast growth factor
Bcl-xL	B-cell lymphoma-extra large
CAF	cancer-associated fibroblast
DDR	discoidin domain receptor
ECM	extracellular matrix
EGF	epidermal growth factor
EGFR-TKI	epidermal growth factor receptor tyrosine kinase inhibitors
EMT	epithelial-to-mesenchymal transition
ERK	extracellular signal-regulated kinases
EV	extracellular vesicles
FAK	focal adhesion kinase
LOX	lysyloxidases
MBV	matrix-bound nanovesicles
MMP	metalloproteinase
PDGF	platelet-derived growth factor
ROS	reactive oxygen species
TACS	tumor-associated collagen signatures
TGF-β	transforming growth factor
TNF-α	tumor necrosis factor alpha
uPA	urokinase-type plasminogen activator
VEGF	vascular endothelial growth factor

## References

[1] Rowe R.G., Weiss S.J. (2009). Navigating ECM Barriers at the Invasive Front: The Cancer Cell-Stroma Interface. Annu. Rev. Cell Dev. Biol..

[2] Piperigkou Z., Kyriakopoulou K., Koutsakis C., Mastronikolis S., Karamanos N.K. (2021). Key Matrix Remodeling Enzymes: Functions and Targeting in Cancer. Cancers.

[3] Ye M., Song Y., Pan S., Chu M., Wang Z.-W., Zhu X. (2020). Evolving Roles of Lysyl Oxidase Family in Tumorigenesis and Cancer Therapy. Pharmacol. Ther..

[4] Dong Q., Liu X., Cheng K., Sheng J., Kong J., Liu T. (2021). Pre-Metastatic Niche Formation in Different Organs Induced by Tumor Extracellular Vesicles. Front. Cell Dev. Biol..

[5] He C., Wang L., Li L., Zhu G. (2021). Extracellular Vesicle-Orchestrated Crosstalk between Cancer-Associated Fibroblasts and Tumors. Transl. Oncol..

[6] Manou D., Caon I., Bouris P., Triantaphyllidou I.-E., Giaroni C., Passi A., Karamanos N.K., Vigetti D., Theocharis A.D. (2019). The Complex Interplay between Extracellular Matrix and Cells in Tissues. Methods Mol. Biol..

[7] Niland S., Eble J.A. (2020). Hold on or Cut? Integrin- and MMP-Mediated Cell-Matrix Interactions in the Tumor Microenvironment. Int. J. Mol. Sci..

[8] Theocharis A.D., Skandalis S.S., Gialeli C., Karamanos N.K. (2016). Extracellular Matrix Structure. Adv. Drug Deliv. Rev..

[9] Karamanos N.K., Theocharis A.D., Piperigkou Z., Manou D., Passi A., Skandalis S.S., Vynios D.H., Orian-Rousseau V., Ricard-Blum S., Schmelzer C.E.H. (2021). A Guide to the Composition and Functions of the Extracellular Matrix. FEBS J..

[10] Jayadev R., Sherwood D.R. (2017). Basement Membranes. Curr. Biol..

[11] Yurchenco P.D. (2011). Basement Membranes: Cell Scaffoldings and Signaling Platforms. Cold Spring Harb. Perspect. Biol..

[12] Pozzi A., Yurchenco P.D., Iozzo R.V. (2017). The Nature and Biology of Basement Membranes. Matrix Biol..

[13] Ricard-Blum S. (2011). The Collagen Family. Cold Spring Harb. Perspect. Biol..

[14] Khoshnoodi J., Pedchenko V., Hudson B.G. (2008). Mammalian Collagen IV. Microsc. Res. Tech..

[15] Aumailley M. (2013). The Laminin Family. Cell Adh. Migr..

[16] Aumailley M., Bruckner-Tuderman L., Carter W.G., Deutzmann R., Edgar D., Ekblom P., Engel J., Engvall E., Hohenester E., Jones J.C.R. (2005). A Simplified Laminin Nomenclature. Matrix Biol..

[17] Hohenester E., Yurchenco P.D. (2013). Laminins in Basement Membrane Assembly. Cell Adh. Migr..

[18] Theocharis A.D., Manou D., Karamanos N.K. (2019). The Extracellular Matrix as a Multitasking Player in Disease. FEBS J..

[19] Miner J.H., Li C., Mudd J.L., Go G., Sutherland A.E. (2004). Compositional and Structural Requirements for Laminin and Basement Membranes during Mouse Embryo Implantation and Gastrulation. Development.

[20] Pöschl E., Schlötzer-Schrehardt U., Brachvogel B., Saito K., Ninomiya Y., Mayer U. (2004). Collagen IV Is Essential for Basement Membrane Stability but Dispensable for Initiation of Its Assembly during Early Development. Development.

[21] Hohenester E. (2019). Structural Biology of Laminins. Essays Biochem..

[22] LeBleu V.S., Macdonald B., Kalluri R. (2007). Structure and Function of Basement Membranes. Exp. Biol. Med..

[23] Gerarduzzi C., Hartmann U., Leask A., Drobetsky E. (2020). The Matrix Revolution: Matricellular Proteins and Restructuring of the Cancer Microenvironment. Cancer Res..

[24] Murphy-Ullrich J.E., Sage E.H. (2014). Revisiting the Matricellular Concept. Matrix Biol..

[25] Iozzo R.V., Schaefer L. (2015). Proteoglycan Form and Function: A Comprehensive Nomenclature of Proteoglycans. Matrix Biol..

[26] Mecham R.P. (2012). Overview of Extracellular Matrix. Current Protocols in Cell Biology.

[27] Bachmann M., Kukkurainen S., Hytönen V.P., Wehrle-Haller B. (2019). Cell Adhesion by Integrins. Physiol. Rev..

[28] Kadry Y.A., Calderwood D.A. (2020). Chapter 22: Structural and Signaling Functions of Integrins. Biochim. Biophys. Acta Biomembr..

[29] Hynes R.O. (2002). Integrins: Bidirectional, Allosteric Signaling Machines. Cell.

[30] Harburger D.S., Calderwood D.A. (2009). Integrin Signalling at a Glance. J. Cell Sci..

[31] Cooper J., Giancotti F.G. (2019). Integrin Signaling in Cancer: Mechanotransduction, Stemness, Epithelial Plasticity, and Therapeutic Resistance. Cancer Cell..

[32] Hamidi H., Ivaska J. (2018). Every Step of the Way: Integrins in Cancer Progression and Metastasis. Nat. Rev. Cancer.

[33] Brittingham R., Uitto J., Fertala A. (2006). High-Affinity Binding of the NC1 Domain of Collagen VII to Laminin 5 and Collagen IV. Biochem. Biophys. Res. Commun..

[34] Chen M., Marinkovich M.P., Veis A., Cai X., Rao C.N., O’Toole E.A., Woodley D.T. (1997). Interactions of the Amino-Terminal Noncollagenous (NC1) Domain of Type VII Collagen with Extracellular Matrix Components: A potential role in epidermal-dermal adherence in human skin. J. Biol. Chem..

[35] Rousselle P., Keene D.R., Ruggiero F., Champliaud M.F., Rest M., Burgeson R.E. (1997). Laminin 5 Binds the NC-1 Domain of Type VII Collagen. J. Cell Biol..

[36] Plikus M.V., Wang X., Sinha S., Forte E., Thompson S.M., Herzog E.L., Driskell R.R., Rosenthal N., Biernaskie J., Horsley V. (2021). Fibroblasts: Origins, Definitions, and Functions in Health and Disease. Cell.

[37] Clark R.A., Lanigan J.M., DellaPelle P., Manseau E., Dvorak H.F., Colvin R.B. (1982). Fibronectin and Fibrin Provide a Provisional Matrix for Epidermal Cell Migration during Wound Reepithelialization. J. Investig. Dermatol..

[38] Fujikawa L.S., Foster C.S., Gipson I.K., Colvin R.B. (1984). Basement Membrane Components in Healing Rabbit Corneal Epithelial Wounds: Immunofluorescence and Ultrastructural Studies. J. Cell Biol..

[39] Wilkinson H.N., Hardman M.J. (2020). Wound Healing: Cellular Mechanisms and Pathological Outcomes. Open Biol..

[40] Li J., Chen J., Kirsner R. (2007). Pathophysiology of Acute Wound Healing. Clin. Dermatol..

[41] Barker T.H., Engler A.J. (2017). The Provisional Matrix: Setting the Stage for Tissue Repair Outcomes. Matrix Biol..

[42] Desmoulière A., Redard M., Darby I., Gabbiani G. (1995). Apoptosis Mediates the Decrease in Cellularity during the Transition between Granulation Tissue and Scar. Am. J. Pathol..

[43] Sahai E., Astsaturov I., Cukierman E., DeNardo D.G., Egeblad M., Evans R.M., Fearon D., Greten F.R., Hingorani S.R., Hunter T. (2020). A Framework for Advancing Our Understanding of Cancer-Associated Fibroblasts. Nat. Rev. Cancer.

[44] Caja L., Dituri F., Mancarella S., Caballero-Diaz D., Moustakas A., Giannelli G., Fabregat I. (2018). TGF-β and the Tissue Microenvironment: Relevance in Fibrosis and Cancer. Int. J. Mol. Sci..

[45] Chung J.Y.-F., Chan M.K.-K., Li J.S.-F., Chan A.S.-W., Tang P.C.-T., Leung K.-T., To K.-F., Lan H.-Y., Tang P.M.-K. (2021). TGF-β Signaling: From Tissue Fibrosis to Tumor Microenvironment. Int. J. Mol. Sci..

[46] Hawinkels L.J.A.C., Paauwe M., Verspaget H.W., Wiercinska E., van der Zon J.M., van der Ploeg K., Koelink P.J., Lindeman J.H.N., Mesker W., Ten Dijke P. (2014). Interaction with Colon Cancer Cells Hyperactivates TGF-β Signaling in Cancer-Associated Fibroblasts. Oncogene.

[47] Ringuette Goulet C., Bernard G., Tremblay S., Chabaud S., Bolduc S., Pouliot F. (2018). Exosomes Induce Fibroblast Differentiation into Cancer-Associated Fibroblasts through TGFβ Signaling. Mol. Cancer Res..

[48] Untergasser G., Gander R., Lilg C., Lepperdinger G., Plas E., Berger P. (2005). Profiling Molecular Targets of TGF-Beta1 in Prostate Fibroblast-to-Myofibroblast Transdifferentiation. Mech. Ageing Dev..

[49] Watt D.M., Morton J.P. (2021). Heterogeneity in Pancreatic Cancer Fibroblasts-TGFβ as a Master Regulator?. Cancers.

[50] Yu Y., Xiao C.-H., Tan L.-D., Wang Q.-S., Li X.-Q., Feng Y.-M. (2014). Cancer-Associated Fibroblasts Induce Epithelial-Mesenchymal Transition of Breast Cancer Cells through Paracrine TGF-β Signalling. Br. J. Cancer.

[51] Hynes R.O. (2009). The Extracellular Matrix: Not Just Pretty Fibrils. Science.

[52] Öhlund D., Handly-Santana A., Biffi G., Elyada E., Almeida A.S., Ponz-Sarvise M., Corbo V., Oni T.E., Hearn S.A., Lee E.J. (2017). Distinct Populations of Inflammatory Fibroblasts and Myofibroblasts in Pancreatic Cancer. J. Exp. Med..

[53] Bochet L., Lehuédé C., Dauvillier S., Wang Y.Y., Dirat B., Laurent V., Dray C., Guiet R., Maridonneau-Parini I., Le Gonidec S. (2013). Adipocyte-Derived Fibroblasts Promote Tumor Progression and Contribute to the Desmoplastic Reaction in Breast Cancer. Cancer Res..

[54] Quante M., Tu S.P., Tomita H., Gonda T., Wang S.S.W., Takashi S., Baik G.H., Shibata W., Diprete B., Betz K.S. (2011). Bone Marrow-Derived Myofibroblasts Contribute to the Mesenchymal Stem Cell Niche and Promote Tumor Growth. Cancer Cell..

[55] Shangguan L., Ti X., Krause U., Hai B., Zhao Y., Yang Z., Liu F. (2012). Inhibition of TGF-β/Smad Signaling by BAMBI Blocks Differentiation of Human Mesenchymal Stem Cells to Carcinoma-Associated Fibroblasts and Abolishes Their Protumor Effects. Stem Cells.

[56] McDonald L.T., LaRue A.C. (2012). Hematopoietic Stem Cell Derived Carcinoma-Associated Fibroblasts: A Novel Origin. Int. J. Clin. Exp. Pathol..

[57] Ogawa M., LaRue A.C., Drake C.J. (2006). Hematopoietic Origin of Fibroblasts/Myofibroblasts: Its Pathophysiologic Implications. Blood.

[58] Zeisberg E.M., Potenta S., Xie L., Zeisberg M., Kalluri R. (2007). Discovery of Endothelial to Mesenchymal Transition as a Source for Carcinoma-Associated Fibroblasts. Cancer Res..

[59] Ganguly D., Chandra R., Karalis J., Teke M., Aguilera T., Maddipati R., Wachsmann M.B., Ghersi D., Siravegna G., Zeh H.J. (2020). Cancer-Associated Fibroblasts: Versatile Players in the Tumor Microenvironment. Cancers.

[60] Sugimoto H., Mundel T.M., Kieran M.W., Kalluri R. (2006). Identification of Fibroblast Heterogeneity in the Tumor Microenvironment. Cancer Biol. Ther..

[61] Nurmik M., Ullmann P., Rodriguez F., Haan S., Letellier E. (2020). In Search of Definitions: Cancer-Associated Fibroblasts and Their Markers. Int. J. Cancer.

[62] Park J.E., Lenter M.C., Zimmermann R.N., Garin-Chesa P., Old L.J., Rettig W.J. (1999). Fibroblast Activation Protein, a Dual Specificity Serine Protease Expressed in Reactive Human Tumor Stromal Fibroblasts. J. Biol. Chem..

[63] Sappino A.P., Skalli O., Jackson B., Schürch W., Gabbiani G. (1988). Smooth-Muscle Differentiation in Stromal Cells of Malignant and Non-Malignant Breast Tissues. Int. J. Cancer.

[64] Strutz F., Okada H., Lo C.W., Danoff T., Carone R.L., Tomaszewski J.E., Neilson E.G. (1995). Identification and Characterization of a Fibroblast Marker: FSP1. J. Cell Biol..

[65] Darby I.A., Laverdet B., Bonté F., Desmoulière A. (2014). Fibroblasts and Myofibroblasts in Wound Healing. Clin. Cosmet. Investig. Dermatol..

[66] Darby I.A., Hewitson T.D. (2007). Fibroblast Differentiation in Wound Healing and Fibrosis. Int. Rev. Cytol..

[67] Hinz B., Gabbiani G. (2003). Cell-Matrix and Cell-Cell Contacts of Myofibroblasts: Role in Connective Tissue Remodeling. Thromb. Haemost..

[68] Attieh Y., Clark A.G., Grass C., Richon S., Pocard M., Mariani P., Elkhatib N., Betz T., Gurchenkov B., Vignjevic D.M. (2017). Cancer-Associated Fibroblasts Lead Tumor Invasion through Integrin-Β3-Dependent Fibronectin Assembly. J. Cell Biol..

[69] Erdogan B., Ao M., White L.M., Means A.L., Brewer B.M., Yang L., Washington M.K., Shi C., Franco O.E., Weaver A.M. (2017). Cancer-Associated Fibroblasts Promote Directional Cancer Cell Migration by Aligning Fibronectin. J. Cell Biol..

[70] Socovich A.M., Naba A. (2019). The Cancer Matrisome: From Comprehensive Characterization to Biomarker Discovery. Semin. Cell Dev. Biol..

[71] Čunderlíková B. (2016). Clinical Significance of Immunohistochemically Detected Extracellular Matrix Proteins and Their Spatial Distribution in Primary Cancer. Crit. Rev. Oncol. Hematol..

[72] Erdogan B., Webb D.J. (2017). Cancer-Associated Fibroblasts Modulate Growth Factor Signaling and Extracellular Matrix Remodeling to Regulate Tumor Metastasis. Biochem. Soc. Trans..

[73] Fang S., Dai Y., Mei Y., Yang M., Hu L., Yang H., Guan X., Li J. (2019). Clinical Significance and Biological Role of Cancer-Derived Type I Collagen in Lung and Esophageal Cancers. Thorac. Cancer.

[74] Öhlund D., Franklin O., Lundberg E., Lundin C., Sund M. (2013). Type IV Collagen Stimulates Pancreatic Cancer Cell Proliferation, Migration, and Inhibits Apoptosis through an Autocrine Loop. BMC Cancer.

[75] Naba A., Clauser K.R., Lamar J.M., Carr S.A., Hynes R.O. (2014). Extracellular Matrix Signatures of Human Mammary Carcinoma Identify Novel Metastasis Promoters. Elife.

[76] Naba A., Clauser K.R., Hoersch S., Liu H., Carr S.A., Hynes R.O. (2012). The Matrisome: In Silico Definition and in Vivo Characterization by Proteomics of Normal and Tumor Extracellular Matrices. Mol. Cell Proteom..

[77] Hebert J.D., Myers S.A., Naba A., Abbruzzese G., Lamar J.M., Carr S.A., Hynes R.O. (2020). Proteomic Profiling of the ECM of Xenograft Breast Cancer Metastases in Different Organs Reveals Distinct Metastatic Niches. Cancer Res..

[78] Tian C., Clauser K.R., Öhlund D., Rickelt S., Huang Y., Gupta M., Mani D.R., Carr S.A., Tuveson D.A., Hynes R.O. (2019). Proteomic Analyses of ECM during Pancreatic Ductal Adenocarcinoma Progression Reveal Different Contributions by Tumor and Stromal Cells. Proc. Natl. Acad. Sci. USA.

[79] Tian C., Öhlund D., Rickelt S., Lidström T., Huang Y., Hao L., Zhao R.T., Franklin O., Bhatia S.N., Tuveson D.A. (2020). Cancer Cell-Derived Matrisome Proteins Promote Metastasis in Pancreatic Ductal Adenocarcinoma. Cancer Res..

[80] Best S.L., Liu Y., Keikhosravi A., Drifka C.R., Woo K.M., Mehta G.S., Altwegg M., Thimm T.N., Houlihan M., Bredfeldt J.S. (2019). Collagen Organization of Renal Cell Carcinoma Differs between Low and High Grade Tumors. BMC Cancer.

[81] Conklin M.W., Eickhoff J.C., Riching K.M., Pehlke C.A., Eliceiri K.W., Provenzano P.P., Friedl A., Keely P.J. (2011). Aligned Collagen Is a Prognostic Signature for Survival in Human Breast Carcinoma. Am. J. Pathol..

[82] Provenzano P.P., Eliceiri K.W., Campbell J.M., Inman D.R., White J.G., Keely P.J. (2006). Collagen Reorganization at the Tumor-Stromal Interface Facilitates Local Invasion. BMC Med..

[83] Brauchle E., Kasper J., Daum R., Schierbaum N., Falch C., Kirschniak A., Schäffer T.E., Schenke-Layland K. (2018). Biomechanical and Biomolecular Characterization of Extracellular Matrix Structures in Human Colon Carcinomas. Matrix Biol..

[84] Zhou Z.-H., Ji C.-D., Xiao H.-L., Zhao H.-B., Cui Y.-H., Bian X.-W. (2017). Reorganized Collagen in the Tumor Microenvironment of Gastric Cancer and Its Association with Prognosis. J. Cancer.

[85] Fleszar A.J., Walker A., Porubsky V., Flanigan W., James D., Campagnola P.J., Weisman P.S., Kreeger P.K. (2018). The Extracellular Matrix of Ovarian Cortical Inclusion Cysts Modulates Invasion of Fallopian Tube Epithelial Cells. APL Bioeng..

[86] Nadiarnykh O., LaComb R.B., Brewer M.A., Campagnola P.J. (2010). Alterations of the Extracellular Matrix in Ovarian Cancer Studied by Second Harmonic Generation Imaging Microscopy. BMC Cancer.

[87] Cicchi R., Massi D., Sestini S., Carli P., Giorgi V.D., Lotti T., Pavone F.S. (2007). Multidimensional Non-Linear Laser Imaging of Basal Cell Carcinoma. Opt. Express..

[88] Keikhosravi A., Shribak M., Conklin M.W., Liu Y., Li B., Loeffler A., Levenson R.M., Eliceiri K.W. (2021). Real-Time Polarization Microscopy of Fibrillar Collagen in Histopathology. Sci. Rep..

[89] Pointer K.B., Clark P.A., Schroeder A.B., Salamat M.S., Eliceiri K.W., Kuo J.S. (2017). Association of Collagen Architecture with Glioblastoma Patient Survival. J. Neurosurg..

[90] Drifka C.R., Loeffler A.G., Mathewson K., Keikhosravi A., Eickhoff J.C., Liu Y., Weber S.M., Kao W.J., Eliceiri K.W. (2016). Highly Aligned Stromal Collagen Is a Negative Prognostic Factor Following Pancreatic Ductal Adenocarcinoma Resection. Oncotarget.

[91] Drifka C.R., Tod J., Loeffler A.G., Liu Y., Thomas G.J., Eliceiri K.W., Kao W.J. (2015). Periductal Stromal Collagen Topology of Pancreatic Ductal Adenocarcinoma Differs from That of Normal and Chronic Pancreatitis. Mod. Pathol..

[92] Provenzano P.P., Inman D.R., Eliceiri K.W., Knittel J.G., Yan L., Rueden C.T., White J.G., Keely P.J. (2008). Collagen Density Promotes Mammary Tumor Initiation and Progression. BMC Med..

[93] Zhang K., Corsa C.A., Ponik S.M., Prior J.L., Piwnica-Worms D., Eliceiri K.W., Keely P.J., Longmore G.D. (2013). The Collagen Receptor Discoidin Domain Receptor 2 Stabilizes SNAIL1 to Facilitate Breast Cancer Metastasis. Nat. Cell Biol..

[94] Chen W., Dong S., Liu X., Wang G., Xu S., Lei S., Zhuo S., Yan J. (2021). Association of the Collagen Signature in the Tumor Microenvironment With Recurrence and Survival of Patients With T4N0M0 Colon Cancer. Dis. Colon Rectum.

[95] Hanley C.J., Noble F., Ward M., Bullock M., Drifka C., Mellone M., Manousopoulou A., Johnston H.E., Hayden A., Thirdborough S. (2016). A Subset of Myofibroblastic Cancer-Associated Fibroblasts Regulate Collagen Fiber Elongation, Which Is Prognostic in Multiple Cancers. Oncotarget.

[96] Ray A., Provenzano P.P. (2021). Aligned Forces: Origins and Mechanisms of Cancer Dissemination Guided by Extracellular Matrix Architecture. Curr. Opin. Cell Biol..

[97] Dumont N., Liu B., Defilippis R.A., Chang H., Rabban J.T., Karnezis A.N., Tjoe J.A., Marx J., Parvin B., Tlsty T.D. (2013). Breast Fibroblasts Modulate Early Dissemination, Tumorigenesis, and Metastasis through Alteration of Extracellular Matrix Characteristics. Neoplasia.

[98] Levental K.R., Yu H., Kass L., Lakins J.N., Egeblad M., Erler J.T., Fong S.F.T., Csiszar K., Giaccia A., Weninger W. (2009). Matrix Crosslinking Forces Tumor Progression by Enhancing Integrin Signaling. Cell.

[99] Chen W.T. (1989). Proteolytic Activity of Specialized Surface Protrusions Formed at Rosette Contact Sites of Transformed Cells. J. Exp. Zool..

[100] Artym V.V., Swatkoski S., Matsumoto K., Campbell C.B., Petrie R.J., Dimitriadis E.K., Li X., Mueller S.C., Bugge T.H., Gucek M. (2015). Dense Fibrillar Collagen Is a Potent Inducer of Invadopodia via a Specific Signaling Network. J. Cell Biol..

[101] Juin A., Billottet C., Moreau V., Destaing O., Albiges-Rizo C., Rosenbaum J., Génot E., Saltel F. (2012). Physiological Type I Collagen Organization Induces the Formation of a Novel Class of Linear Invadosomes. Mol. Biol. Cell.

[102] Yan T., Zhang A., Shi F., Chang F., Mei J., Liu Y., Zhu Y. (2018). Integrin Avβ3-Associated DAAM1 Is Essential for Collagen-Induced Invadopodia Extension and Cell Haptotaxis in Breast Cancer Cells. J. Biol. Chem..

[103] Azemikhah M., Ashtiani H.A., Aghaei M., Rastegar H. (2015). Evaluation of Discoidin Domain Receptor-2 (DDR2) Expression Level in Normal, Benign, and Malignant Human Prostate Tissues. Res. Pharm. Sci..

[104] Hur H., Ham I.-H., Lee D., Jin H., Aguilera K.Y., Oh H.J., Han S.-U., Kwon J.E., Kim Y.-B., Ding K. (2017). Discoidin Domain Receptor 1 Activity Drives an Aggressive Phenotype in Gastric Carcinoma. BMC Cancer.

[105] Jin H., Ham I.-H., Oh H.J., Bae C.A., Lee D., Kim Y.-B., Son S.-Y., Chwae Y.-J., Han S.-U., Brekken R.A. (2018). Inhibition of Discoidin Domain Receptor 1 Prevents Stroma-Induced Peritoneal Metastasis in Gastric Carcinoma. Mol. Cancer Res..

[106] Romayor I., Badiola I., Benedicto A., Márquez J., Herrero A., Arteta B., Olaso E. (2020). Silencing of Sinusoidal DDR1 Reduces Murine Liver Metastasis by Colon Carcinoma. Sci. Rep..

[107] Gadiya M., Chakraborty G. (2018). Signaling by Discoidin Domain Receptor 1 in Cancer Metastasis. Cell Adh. Migr..

[108] Majo S., Auguste P. (2021). The Yin and Yang of Discoidin Domain Receptors (DDRs): Implications in Tumor Growth and Metastasis Development. Cancers.

[109] Mehta V., Chander H., Munshi A. (2021). Complex Roles of Discoidin Domain Receptor Tyrosine Kinases in Cancer. Clin. Transl. Oncol..

[110] Nissen N.I., Karsdal M., Willumsen N. (2019). Collagens and Cancer Associated Fibroblasts in the Reactive Stroma and Its Relation to Cancer Biology. J. Exp. Clin. Cancer Res..

[111] van Huizen N.A., Coebergh van den Braak R.R.J., Doukas M., Dekker L.J.M., IJzermans J.N.M., Luider T.M. (2019). Up-Regulation of Collagen Proteins in Colorectal Liver Metastasis Compared with Normal Liver Tissue. J. Biol. Chem..

[112] Bourgot I., Primac I., Louis T., Noël A., Maquoi E. (2020). Reciprocal Interplay between Fibrillar Collagens and Collagen-Binding Integrins: Implications in Cancer Progression and Metastasis. Front. Oncol..

[113] Ferreira A.R., Alho I., Shan N., Matias M., Faria M., Casimiro S., Leitzel K., Ali S., Lipton A., Costa L. (2016). N-Telopeptide of Type I Collagen Long-Term Dynamics in Breast Cancer Patients with Bone Metastases: Clinical Outcomes and Influence of Extraskeletal Metastases. Oncologist.

[114] Hall C.L., Dai J., van Golen K.L., Keller E.T., Long M.W. (2006). Type I Collagen Receptor (Alpha 2 Beta 1) Signaling Promotes the Growth of Human Prostate Cancer Cells within the Bone. Cancer Res..

[115] Brooks M., Mo Q., Krasnow R., Ho P.L., Lee Y.-C., Xiao J., Kurtova A., Lerner S., Godoy G., Jian W. (2016). Positive Association of Collagen Type I with Non-Muscle Invasive Bladder Cancer Progression. Oncotarget.

[116] Huang C., Yang X., Han L., Fan Z., Liu B., Zhang C., Lu T. (2019). The Prognostic Potential of Alpha-1 Type I Collagen Expression in Papillary Thyroid Cancer. Biochem. Biophys. Res. Commun..

[117] Hou L., Lin T., Wang Y., Liu B., Wang M. (2021). Collagen Type 1 Alpha 1 Chain Is a Novel Predictive Biomarker of Poor Progression-Free Survival and Chemoresistance in Metastatic Lung Cancer. J. Cancer.

[118] Barcus C.E., O’Leary K.A., Brockman J.L., Rugowski D.E., Liu Y., Garcia N., Yu M., Keely P.J., Eliceiri K.W., Schuler L.A. (2017). Elevated Collagen-I Augments Tumor Progressive Signals, Intravasation and Metastasis of Prolactin-Induced Estrogen Receptor Alpha Positive Mammary Tumor Cells. Breast. Cancer Res..

[119] Yamazaki S., Higuchi Y., Ishibashi M., Hashimoto H., Yasunaga M., Matsumura Y., Tsuchihara K., Tsuboi M., Goto K., Ochiai A. (2018). Collagen Type I Induces EGFR-TKI Resistance in EGFR-Mutated Cancer Cells by MTOR Activation through Akt-Independent Pathway. Cancer Sci..

[120] Yamazaki S., Su Y., Maruyama A., Makinoshima H., Suzuki J., Tsuboi M., Goto K., Ochiai A., Ishii G. (2020). Uptake of Collagen Type I via Macropinocytosis Cause MTOR Activation and Anti-Cancer Drug Resistance. Biochem. Biophys. Res. Commun..

[121] Nah H.D., Barembaum M., Upholt W.B. (1992). The Chicken Alpha 1 (XI) Collagen Gene Is Widely Expressed in Embryonic Tissues. J. Biol. Chem..

[122] Mendler M., Eich-Bender S.G., Vaughan L., Winterhalter K.H., Bruckner P. (1989). Cartilage Contains Mixed Fibrils of Collagen Types II, IX, and XI. J. Cell Biol..

[123] Jia D., Liu Z., Deng N., Tan T.Z., Huang R.Y.-J., Taylor-Harding B., Cheon D.-J., Lawrenson K., Wiedemeyer W.R., Walts A.E. (2016). A COL11A1-Correlated Pan-Cancer Gene Signature of Activated Fibroblasts for the Prioritization of Therapeutic Targets. Cancer Lett..

[124] García-Pravia C., Galván J.A., Gutiérrez-Corral N., Solar-García L., García-Pérez E., García-Ocaña M., Del Amo-Iribarren J., Menéndez-Rodríguez P., García-García J., de Los Toyos J.R. (2013). Overexpression of COL11A1 by Cancer-Associated Fibroblasts: Clinical Relevance of a Stromal Marker in Pancreatic Cancer. PLoS ONE.

[125] Cheon D.-J., Tong Y., Sim M.-S., Dering J., Berel D., Cui X., Lester J., Beach J.A., Tighiouart M., Walts A.E. (2014). A Collagen-Remodeling Gene Signature Regulated by TGF-β Signaling Is Associated with Metastasis and Poor Survival in Serous Ovarian Cancer. Clin. Cancer Res..

[126] Wu Y.-H., Chang T.-H., Huang Y.-F., Huang H.-D., Chou C.-Y. (2014). COL11A1 Promotes Tumor Progression and Predicts Poor Clinical Outcome in Ovarian Cancer. Oncogene.

[127] Wu Y.-H., Huang Y.-F., Chang T.-H., Chen C.-C., Wu P.-Y., Huang S.-C., Chou C.-Y. (2021). COL11A1 Activates Cancer-Associated Fibroblasts by Modulating TGF-Β3 through the NF-ΚB/IGFBP2 Axis in Ovarian Cancer Cells. Oncogene.

[128] Raglow Z., Thomas S.M. (2015). Tumor Matrix Protein Collagen XIα1 in Cancer. Cancer Lett..

[129] Liu Z., Lai J., Jiang H., Ma C., Huang H. (2021). Collagen XI Alpha 1 Chain, a Potential Therapeutic Target for Cancer. FASEB J..

[130] Lee C.S., Siprashvili Z., Mah A., Bencomo T., Elcavage L.E., Che Y., Shenoy R.M., Aasi S.Z., Khavari P.A. (2021). Mutant Collagen COL11A1 Enhances Cancerous Invasion. Oncogene.

[131] Nallanthighal S., Heiserman J.P., Cheon D.-J. (2021). Collagen Type XI Alpha 1 (COL11A1): A Novel Biomarker and a Key Player in Cancer. Cancers.

[132] Wang H., Ren R., Yang Z., Cai J., Du S., Shen X. (2021). The COL11A1/Akt/CREB Signaling Axis Enables Mitochondrial-Mediated Apoptotic Evasion to Promote Chemoresistance in Pancreatic Cancer Cells through Modulating BAX/BCL-2 Function. J. Cancer.

[133] Nissen N.I., Kehlet S., Johansen A.Z., Chen I.M., Karsdal M., Johansen J.S., Diab H.M.H., Jørgensen L.N., Sun S., Manon-Jensen T. (2021). Noninvasive Prognostic Biomarker Potential of Quantifying the Propeptides of Type XI Collagen Alpha-1 Chain (PRO-C11) in Patients with Pancreatic Ductal Adenocarcinoma. Int. J. Cancer.

[134] Wang J., Jiang Y.-H., Yang P.-Y., Liu F. (2021). Increased Collagen Type V A2 (COL5A2) in Colorectal Cancer Is Associated with Poor Prognosis and Tumor Progression. OncoTargets Ther..

[135] Huang G., Ge G., Izzi V., Greenspan D.S. (2017). A3 Chains of Type V Collagen Regulate Breast Tumour Growth via Glypican-1. Nat. Commun..

[136] Chen H.-C., Tseng Y.-K., Shu C.-W., Weng T.-J., Liou H.-H., Yen L.-M., Hsieh I.-C., Wang C.-C., Wu P.-C., Shiue Y.-L. (2019). Differential Clinical Significance of COL5A1 and COL5A2 in Tongue Squamous Cell Carcinoma. J. Oral. Pathol. Med..

[137] Tan Y., Chen Q., Xing Y., Zhang C., Pan S., An W., Xu H. (2021). High Expression of COL5A2, a Member of COL5 Family, Indicates the Poor Survival and Facilitates Cell Migration in Gastric Cancer. Biosci. Rep..

[138] Cescon M., Gattazzo F., Chen P., Bonaldo P. (2015). Collagen VI at a Glance. J. Cell Sci..

[139] Williams L., Layton T., Yang N., Feldmann M., Nanchahal J. (2021). Collagen VI as a Driver and Disease Biomarker in Human Fibrosis. FEBS J..

[140] Iyengar P., Espina V., Williams T.W., Lin Y., Berry D., Jelicks L.A., Lee H., Temple K., Graves R., Pollard J. (2005). Adipocyte-Derived Collagen VI Affects Early Mammary Tumor Progression In Vivo, Demonstrating a Critical Interaction in the Tumor/Stroma Microenvironment. J. Clin. Investig..

[141] Chen P., Cescon M., Bonaldo P. (2013). Collagen VI in Cancer and Its Biological Mechanisms. Trends. Mol. Med..

[142] Ho C.-M., Chang T.-H., Yen T.-L., Hong K.-J., Huang S.-H. (2021). Collagen Type VI Regulates the CDK4/6-p-Rb Signaling Pathway and Promotes Ovarian Cancer Invasiveness, Stemness, and Metastasis. Am. J. Cancer Res..

[143] Duan Y., Liu G., Sun Y., Wu J., Xiong Z., Jin T., Chen M. (2020). Collagen Type VI A5 Gene Variations May Predict the Risk of Lung Cancer Development in Chinese Han Population. Sci. Rep..

[144] Sato T., Tokunaka K., Saiga K., Tomura A., Sugihara H., Hayashi T., Imamura Y., Morita M. (2020). Involvement of Non-triple Helical Type VI Collagen A1 Chain, NTH A1(VI), in the Proliferation of Cancer Cells. Oncol. Rep..

[145] Owusu-Ansah K.G., Song G., Chen R., Edoo M.I.A., Li J., Chen B., Wu J., Zhou L., Xie H., Jiang D. (2019). COL6A1 Promotes Metastasis and Predicts Poor Prognosis in Patients with Pancreatic Cancer. Int. J. Oncol..

[146] Park J., Scherer P.E. (2012). Adipocyte-Derived Endotrophin Promotes Malignant Tumor Progression. J. Clin. Investig..

[147] Wang J., Pan W. (2020). The Biological Role of the Collagen Alpha-3 (VI) Chain and Its Cleaved C5 Domain Fragment Endotrophin in Cancer. OncoTargets. Ther..

[148] Bu D., Crewe C., Kusminski C.M., Gordillo R., Ghaben A.L., Kim M., Park J., Deng H., Xiong W., Liu X.-Z. (2019). Human Endotrophin as a Driver of Malignant Tumor Growth. JCI Insight.

[149] Kim M.-S., Ha S.-E., Wu M., Zogg H., Ronkon C.F., Lee M.-Y., Ro S. (2021). Extracellular Matrix Biomarkers in Colorectal Cancer. Int. J. Mol. Sci..

[150] Qin Y., Rodin S., Simonson O.E., Hollande F. (2017). Laminins and Cancer Stem Cells: Partners in Crime?. Semin. Cancer Biol..

[151] Maltseva D.V., Rodin S.A. (2018). Laminins in Metastatic Cancer. Mol. Biol..

[152] Rousselle P., Scoazec J.Y. (2020). Laminin 332 in Cancer: When the Extracellular Matrix Turns Signals from Cell Anchorage to Cell Movement. Semin. Cancer Biol..

[153] Zahir N., Lakins J.N., Russell A., Ming W., Chatterjee C., Rozenberg G.I., Marinkovich M.P., Weaver V.M. (2003). Autocrine Laminin-5 Ligates Alpha6beta4 Integrin and Activates RAC and NFkappaB to Mediate Anchorage-Independent Survival of Mammary Tumors. J. Cell Biol..

[154] Manohar A., Shome S.G., Lamar J., Stirling L., Iyer V., Pumiglia K., DiPersio C.M. (2004). Alpha 3 Beta 1 Integrin Promotes Keratinocyte Cell Survival through Activation of a MEK/ERK Signaling Pathway. J. Cell Sci..

[155] Carpenter P.M., Dao A.V., Arain Z.S., Chang M.K., Nguyen H.P., Arain S., Wang-Rodriguez J., Kwon S.-Y., Wilczynski S.P. (2009). Motility Induction in Breast Carcinoma by Mammary Epithelial Laminin 332 (Laminin 5). Mol. Cancer Res..

[156] Ramovs V., Te Molder L., Sonnenberg A. (2017). The Opposing Roles of Laminin-Binding Integrins in Cancer. Matrix Biol..

[157] Kato K., Shiga K., Yamaguchi K., Hata K., Kobayashi T., Miyazaki K., Saijo S., Miyagi T. (2006). Plasma-Membrane-Associated Sialidase (NEU3) Differentially Regulates Integrin-Mediated Cell Proliferation through Laminin- and Fibronectin-Derived Signalling. Biochem. J..

[158] Aoki S., Nakanishi Y., Akimoto S., Moriya Y., Yoshimura K., Kitajima M., Sakamoto M., Hirohashi S. (2002). Prognostic Significance of Laminin-5 Gamma2 Chain Expression in Colorectal Carcinoma: Immunohistochemical Analysis of 103 Cases. Dis. Colon. Rectum..

[159] Ito E., Ozawa S., Kijima H., Kazuno A., Miyako H., Nishi T., Chino O., Shimada H., Tanaka M., Inoue S. (2014). Clinicopathological Significance of Laminin-5γ2 Chain Expression in Superficial Esophageal Cancer. Dis. Esophagus.

[160] Koshikawa N., Giannelli G., Cirulli V., Miyazaki K., Quaranta V. (2000). Role of Cell Surface Metalloprotease MT1-MMP in Epithelial Cell Migration over Laminin-5. J. Cell Biol..

[161] Gilles C., Polette M., Coraux C., Tournier J.M., Meneguzzi G., Munaut C., Volders L., Rousselle P., Birembaut P., Foidart J.M. (2001). Contribution of MT1-MMP and of Human Laminin-5 Gamma2 Chain Degradation to Mammary Epithelial Cell Migration. J. Cell Sci..

[162] Giannelli G., Falk-Marzillier J., Schiraldi O., Stetler-Stevenson W.G., Quaranta V. (1997). Induction of Cell Migration by Matrix Metalloprotease-2 Cleavage of Laminin-5. Science.

[163] Schenk S., Hintermann E., Bilban M., Koshikawa N., Hojilla C., Khokha R., Quaranta V. (2003). Binding to EGF Receptor of a Laminin-5 EGF-like Fragment Liberated during MMP-Dependent Mammary Gland Involution. J. Cell Biol..

[164] Engel J., Odermatt E., Engel A., Madri J.A., Furthmayr H., Rohde H., Timpl R. (1981). Shapes, Domain Organizations and Flexibility of Laminin and Fibronectin, Two Multifunctional Proteins of the Extracellular Matrix. J. Mol. Biol..

[165] Hynes R.O., Yamada K.M. (1982). Fibronectins: Multifunctional Modular Glycoproteins. J. Cell Biol..

[166] Kornblihtt A.R., Umezawa K., Vibe-Pedersen K., Baralle F.E. (1985). Primary Structure of Human Fibronectin: Differential Splicing May Generate at Least 10 Polypeptides from a Single Gene. EMBO J..

[167] Owens R.J., Kornblihtt A.R., Baralle F.E. (1986). Fibronectin, the Generation of Multiple Polypeptides from a Single Gene. Oxf. Surv. Eukaryot. Genes.

[168] Schwarzbauer J.E., Tamkun J.W., Lemischka I.R., Hynes R.O. (1983). Three Different Fibronectin MRNAs Arise by Alternative Splicing within the Coding Region. Cell.

[169] Yamada K.M., Kennedy D.W. (1979). Fibroblast Cellular and Plasma Fibronectins Are Similar but Not Identical. J. Cell Biol..

[170] Dalton C.J., Lemmon C.A. (2021). Fibronectin: Molecular Structure, Fibrillar Structure and Mechanochemical Signaling. Cells.

[171] Tamkun J.W., Hynes R.O. (1983). Plasma Fibronectin Is Synthesized and Secreted by Hepatocytes. J. Biol. Chem..

[172] Mao Y., Schwarzbauer J.E. (2005). Fibronectin Fibrillogenesis, a Cell-Mediated Matrix Assembly Process. Matrix Biol..

[173] To W.S., Midwood K.S. (2011). Plasma and Cellular Fibronectin: Distinct and Independent Functions during Tissue Repair. Fibrogenes. Tissue Repair.

[174] Gopal S., Veracini L., Grall D., Butori C., Schaub S., Audebert S., Camoin L., Baudelet E., Radwanska A., Beghelli-de la Forest Divonne S. (2017). Fibronectin-Guided Migration of Carcinoma Collectives. Nat. Commun..

[175] Rick J.W., Chandra A., Dalle Ore C., Nguyen A.T., Yagnik G., Aghi M.K. (2019). Fibronectin in Malignancy: Cancer-Specific Alterations, Protumoral Effects, and Therapeutic Implications. Semin. Oncol..

[176] Letunic I., Khedkar S., Bork P. (2021). SMART: Recent Updates, New Developments and Status in 2020. Nucleic Acids Res..

[177] Pankov R., Yamada K.M. (2002). Fibronectin at a Glance. J. Cell Sci..

[178] Schwarzbauer J.E., Paul J.I., Hynes R.O. (1985). On the Origin of Species of Fibronectin. Proc. Natl. Acad. Sci. USA.

[179] Vaidya A., Wang H., Qian V., Gilmore H., Lu Z.-R. (2020). Overexpression of Extradomain-B Fibronectin Is Associated with Invasion of Breast Cancer Cells. Cells.

[180] Birchler M.T., Milisavlijevic D., Pfaltz M., Neri D., Odermatt B., Schmid S., Stoeckli S.J. (2003). Expression of the Extra Domain B of Fibronectin, a Marker of Angiogenesis, in Head and Neck Tumors. Laryngoscope.

[181] Kujawa K.A., Zembala-Nożyńska E., Cortez A.J., Kujawa T., Kupryjańczyk J., Lisowska K.M. (2020). Fibronectin and Periostin as Prognostic Markers in Ovarian Cancer. Cells.

[182] Locher R., Erba P.A., Hirsch B., Bombardieri E., Giovannoni L., Neri D., Dürkop H., Menssen H.D. (2014). Abundant in Vitro Expression of the Oncofetal ED-B-Containing Fibronectin Translates into Selective Pharmacodelivery of (131)I-L19SIP in a Prostate Cancer Patient. J. Cancer Res. Clin. Oncol..

[183] Vaidya A., Ayat N., Buford M., Wang H., Shankardass A., Zhao Y., Gilmore H., Wang Z., Lu Z.-R. (2020). Noninvasive Assessment and Therapeutic Monitoring of Drug-Resistant Colorectal Cancer by MR Molecular Imaging of Extradomain-B Fibronectin. Theranostics.

[184] Xiao J., Yang W., Xu B., Zhu H., Zou J., Su C., Rong J., Wang T., Chen Z. (2018). Expression of Fibronectin in Esophageal Squamous Cell Carcinoma and Its Role in Migration. BMC Cancer.

[185] Lin T.-C., Yang C.-H., Cheng L.-H., Chang W.-T., Lin Y.-R., Cheng H.-C. (2019). Fibronectin in Cancer: Friend or Foe. Cells.

[186] Efthymiou G., Saint A., Ruff M., Rekad Z., Ciais D., Van Obberghen-Schilling E. (2020). Shaping Up the Tumor Microenvironment With Cellular Fibronectin. Front. Oncol..

[187] Spada S., Tocci A., Di Modugno F., Nisticò P. (2021). Fibronectin as a Multiregulatory Molecule Crucial in Tumor Matrisome: From Structural and Functional Features to Clinical Practice in Oncology. J. Exp. Clin. Cancer Res..

[188] Topalovski M., Brekken R.A. (2016). Matrix Control of Pancreatic Cancer: New Insights into Fibronectin Signaling. Cancer Lett..

[189] Vaquero E.C., Edderkaoui M., Nam K.J., Gukovsky I., Pandol S.J., Gukovskaya A.S. (2003). Extracellular Matrix Proteins Protect Pancreatic Cancer Cells from Death via Mitochondrial and Nonmitochondrial Pathways. Gastroenterology.

[190] Edderkaoui M., Hong P., Vaquero E.C., Lee J.K., Fischer L., Friess H., Buchler M.W., Lerch M.M., Pandol S.J., Gukovskaya A.S. (2005). Extracellular Matrix Stimulates Reactive Oxygen Species Production and Increases Pancreatic Cancer Cell Survival through 5-Lipoxygenase and NADPH Oxidase. Am. J. Physiol. Gastrointest. Liver Physiol..

[191] Edderkaoui M., Hong P., Lee J.K., Pandol S.J., Gukovskaya A.S. (2007). Insulin-like Growth Factor-I Receptor Mediates the Prosurvival Effect of Fibronectin. J. Biol. Chem..

[192] Han S., Khuri F.R., Roman J. (2006). Fibronectin Stimulates Non-Small Cell Lung Carcinoma Cell Growth through Activation of Akt/Mammalian Target of Rapamycin/S6 Kinase and Inactivation of LKB1/AMP-Activated Protein Kinase Signal Pathways. Cancer Res..

[193] Cao Y., Liu X., Lu W., Chen Y., Wu X., Li M., Wang X.-A., Zhang F., Jiang L., Zhang Y. (2015). Fibronectin Promotes Cell Proliferation and Invasion through MTOR Signaling Pathway Activation in Gallbladder Cancer. Cancer Lett..

[194] Ou Y.-C., Li J.-R., Wang J.-D., Chang C.-Y., Wu C.-C., Chen W.-Y., Kuan Y.-H., Liao S.-L., Lu H.-C., Chen C.-J. (2019). Fibronectin Promotes Cell Growth and Migration in Human Renal Cell Carcinoma Cells. Int. J. Mol. Sci..

[195] Han S., Sidell N., Roman J. (2005). Fibronectin Stimulates Human Lung Carcinoma Cell Proliferation by Suppressing P21 Gene Expression via Signals Involving Erk and Rho Kinase. Cancer Lett..

[196] Zhong C., Tao B., Tang F., Yang X., Peng T., You J., Xia K., Xia X., Chen L., Peng L. (2021). Remodeling Cancer Stemness by Collagen/Fibronectin via the AKT and CDC42 Signaling Pathway Crosstalk in Glioma. Theranostics.

[197] Albacete-Albacete L., Sánchez-Álvarez M., Del Pozo M.A. (2021). Extracellular Vesicles: An Emerging Mechanism Governing the Secretion and Biological Roles of Tenascin-C. Front. Immunol..

[198] Théry C., Witwer K.W., Aikawa E., Alcaraz M.J., Anderson J.D., Andriantsitohaina R., Antoniou A., Arab T., Archer F., Atkin-Smith G.K. (2018). Minimal Information for Studies of Extracellular Vesicles 2018 (MISEV2018): A Position Statement of the International Society for Extracellular Vesicles and Update of the MISEV2014 Guidelines. J. Extracell. Vesicles.

[199] van Niel G., D’Angelo G., Raposo G. (2018). Shedding Light on the Cell Biology of Extracellular Vesicles. Nat. Rev. Mol. Cell Biol..

[200] Huleihel L., Hussey G.S., Naranjo J.D., Zhang L., Dziki J.L., Turner N.J., Stolz D.B., Badylak S.F. (2016). Matrix-Bound Nanovesicles within ECM Bioscaffolds. Sci. Adv..

[201] Urabe F., Patil K., Ramm G.A., Ochiya T., Soekmadji C. (2021). Extracellular Vesicles in the Development of Organ-Specific Metastasis. J. Extracell. Vesicles.

[202] Stefanius K., Servage K., Orth K. (2021). Exosomes in Cancer Development. Curr. Opin. Genet. Dev..

[203] Schubert A., Boutros M. (2021). Extracellular Vesicles and Oncogenic Signaling. Mol. Oncol..

[204] Hussey G.S., Pineda Molina C., Cramer M.C., Tyurina Y.Y., Tyurin V.A., Lee Y.C., El-Mossier S.O., Murdock M.H., Timashev P.S., Kagan V.E. (2020). Lipidomics and RNA Sequencing Reveal a Novel Subpopulation of Nanovesicle within Extracellular Matrix Biomaterials. Sci. Adv..

[205] Quijano L.M., Naranjo J.D., El-Mossier S.O., Turner N.J., Pineda Molina C., Bartolacci J., Zhang L., White L., Li H., Badylak S.F. (2020). Matrix-Bound Nanovesicles: The Effects of Isolation Method upon Yield, Purity, and Function. Tissue. Eng. Part. C Methods.

[206] Chanda D., Otoupalova E., Hough K.P., Locy M.L., Bernard K., Deshane J.S., Sanderson R.D., Mobley J.A., Thannickal V.J. (2019). Fibronectin on the Surface of Extracellular Vesicles Mediates Fibroblast Invasion. Am. J. Respir. Cell Mol. Biol..

[207] von Au A., Vasel M., Kraft S., Sens C., Hackl N., Marx A., Stroebel P., Hennenlotter J., Todenhöfer T., Stenzl A. (2013). Circulating Fibronectin Controls Tumor Growth. Neoplasia.

[208] Zhang Y., Lu H., Dazin P., Kapila Y. (2004). Squamous Cell Carcinoma Cell Aggregates Escape Suspension-Induced, P53-Mediated Anoikis: Fibronectin and integrin αv mediate survival signals through focal adhesion kinase. J. Biol. Chem..

[209] Han H.-J., Sung J.Y., Kim S.-H., Yun U.-J., Kim H., Jang E.-J., Yoo H.-E., Hong E.K., Goh S.-H., Moon A. (2021). Fibronectin Regulates Anoikis Resistance via Cell Aggregate Formation. Cancer Lett..

[210] Ghura H., Keimer M., von Au A., Hackl N., Klemis V., Nakchbandi I.A. (2021). Inhibition of Fibronectin Accumulation Suppresses Tumor Growth. Neoplasia.

[211] Chiquet M., Fambrough D.M. (1984). Chick Myotendinous Antigen. I. A Monoclonal Antibody as a Marker for Tendon and Muscle Morphogenesis. J. Cell Biol..

[212] Erickson H.P., Inglesias J.L. (1984). A Six-Armed Oligomer Isolated from Cell Surface Fibronectin Preparations. Nature.

[213] Grumet M., Hoffman S., Crossin K.L., Edelman G.M. (1985). Cytotactin, an Extracellular Matrix Protein of Neural and Non-Neural Tissues That Mediates Glia-Neuron Interaction. Proc. Natl. Acad. Sci. USA.

[214] Kruse J., Keilhauer G., Faissner A., Timpl R., Schachner M. (1985). The J1 Glycoprotein--a Novel Nervous System Cell Adhesion Molecule of the L2/HNK-1 Family. Nature.

[215] Jones F.S., Jones P.L. (2000). The Tenascin Family of ECM Glycoproteins: Structure, Function, and Regulation during Embryonic Development and Tissue Remodeling. Dev. Dyn..

[216] Chiquet M., Fambrough D.M. (1984). Chick Myotendinous Antigen. II. A Novel Extracellular Glycoprotein Complex Consisting of Large Disulfide-Linked Subunits. J. Cell Biol..

[217] Jones F.S., Hoffman S., Cunningham B.A., Edelman G.M. (1989). A Detailed Structural Model of Cytotactin: Protein Homologies, Alternative RNA Splicing, and Binding Regions. Proc. Natl. Acad. Sci. USA.

[218] Jones F.S., Burgoon M.P., Hoffman S., Crossin K.L., Cunningham B.A., Edelman G.M. (1988). A CDNA Clone for Cytotactin Contains Sequences Similar to Epidermal Growth Factor-like Repeats and Segments of Fibronectin and Fibrinogen. Proc. Natl. Acad. Sci. USA.

[219] Giblin S.P., Midwood K.S. (2015). Tenascin-C: Form versus Function. Cell Adh. Migr..

[220] Siri A., Knäuper V., Veirana N., Caocci F., Murphy G., Zardi L. (1995). Different Susceptibility of Small and Large Human Tenascin-C Isoforms to Degradation by Matrix Metalloproteinases. J. Biol. Chem..

[221] Tucker R.P., Chiquet-Ehrismann R. (2015). Tenascin-C: Its Functions as an Integrin Ligand. Int. J. Biochem. Cell Biol..

[222] Swindle C.S., Tran K.T., Johnson T.D., Banerjee P., Mayes A.M., Griffith L., Wells A. (2001). Epidermal Growth Factor (EGF)-like Repeats of Human Tenascin-C as Ligands for EGF Receptor. J. Cell Biol..

[223] Chiquet-Ehrismann R., Matsuoka Y., Hofer U., Spring J., Bernasconi C., Chiquet M. (1991). Tenascin Variants: Differential Binding to Fibronectin and Distinct Distribution in Cell Cultures and Tissues. Cell Regul..

[224] Chung C.Y., Zardi L., Erickson H.P. (1995). Binding of Tenascin-C to Soluble Fibronectin and Matrix Fibrils. J. Biol. Chem..

[225] Midwood K.S., Chiquet M., Tucker R.P., Orend G. (2016). Tenascin-C at a Glance. J. Cell Sci..

[226] Midwood K.S., Hussenet T., Langlois B., Orend G. (2011). Advances in Tenascin-C Biology. Cell Mol. Life Sci..

[227] Chiquet-Ehrismann R., Mackie E.J., Pearson C.A., Sakakura T. (1986). Tenascin: An Extracellular Matrix Protein Involved in Tissue Interactions during Fetal Development and Oncogenesis. Cell.

[228] Chiquet-Ehrismann R., Orend G., Chiquet M., Tucker R.P., Midwood K.S. (2014). Tenascins in Stem Cell Niches. Matrix Biol..

[229] Chiovaro F., Chiquet-Ehrismann R., Chiquet M. (2015). Transcriptional Regulation of Tenascin Genes. Cell Adh. Migr..

[230] Scherer C., Pfisterer L., Wagner A.H., Hödebeck M., Cattaruzza M., Hecker M., Korff T. (2014). Arterial Wall Stress Controls NFAT5 Activity in Vascular Smooth Muscle Cells. J. Am. Heart Assoc..

[231] Yamamoto K., Dang Q.N., Kennedy S.P., Osathanondh R., Kelly R.A., Lee R.T. (1999). Induction of Tenascin-C in Cardiac Myocytes by Mechanical Deformation. Role of Reactive Oxygen Species. J. Biol. Chem..

[232] Chiquet-Ehrismann R., Tannheimer M., Koch M., Brunner A., Spring J., Martin D., Baumgartner S., Chiquet M. (1994). Tenascin-C Expression by Fibroblasts Is Elevated in Stressed Collagen Gels. J. Cell Biol..

[233] Jinnin M., Ihn H., Asano Y., Yamane K., Trojanowska M., Tamaki K. (2004). Tenascin-C Upregulation by Transforming Growth Factor-Beta in Human Dermal Fibroblasts Involves Smad3, Sp1, and Ets1. Oncogene.

[234] Pearson C.A., Pearson D., Shibahara S., Hofsteenge J., Chiquet-Ehrismann R. (1988). Tenascin: CDNA Cloning and Induction by TGF-Beta. EMBO J..

[235] Jinnin M., Ihn H., Asano Y., Yamane K., Trojanowska M., Tamaki K. (2006). Platelet Derived Growth Factor Induced Tenascin-C Transcription Is Phosphoinositide 3-Kinase/Akt-Dependent and Mediated by Ets Family Transcription Factors. J. Cell Physiol..

[236] Lowy C.M., Oskarsson T. (2015). Tenascin C in Metastasis: A View from the Invasive Front. Cell Adh. Migr..

[237] Silvers C.R., Messing E.M., Miyamoto H., Lee Y.-F. (2021). Tenascin-C Expression in the Lymph Node Pre-Metastatic Niche in Muscle-Invasive Bladder Cancer. Br. J. Cancer.

[238] Ishihara A., Yoshida T., Tamaki H., Sakakura T. (1995). Tenascin Expression in Cancer Cells and Stroma of Human Breast Cancer and Its Prognostic Significance. Clin. Cancer Res..

[239] Modica C., Olivero M., Zuppini F., Milan M., Basilico C., Vigna E. (2021). HGF/MET Axis Induces Tumor Secretion of Tenascin-C and Promotes Stromal Rewiring in Pancreatic Cancer. Cancers.

[240] O’Connell J.T., Sugimoto H., Cooke V.G., MacDonald B.A., Mehta A.I., LeBleu V.S., Dewar R., Rocha R.M., Brentani R.R., Resnick M.B. (2011). VEGF-A and Tenascin-C Produced by S100A4+ Stromal Cells Are Important for Metastatic Colonization. Proc. Natl. Acad. Sci. USA.

[241] Oskarsson T., Acharyya S., Zhang X.H.-F., Vanharanta S., Tavazoie S.F., Morris P.G., Downey R.J., Manova-Todorova K., Brogi E., Massagué J. (2011). Breast Cancer Cells Produce Tenascin C as a Metastatic Niche Component to Colonize the Lungs. Nat. Med..

[242] Saupe F., Schwenzer A., Jia Y., Gasser I., Spenlé C., Langlois B., Kammerer M., Lefebvre O., Hlushchuk R., Rupp T. (2013). Tenascin-C Downregulates Wnt Inhibitor Dickkopf-1, Promoting Tumorigenesis in a Neuroendocrine Tumor Model. Cell Rep..

[243] Spenlé C., Gasser I., Saupe F., Janssen K.-P., Arnold C., Klein A., van der Heyden M., Mutterer J., Neuville-Méchine A., Chenard M.-P. (2015). Spatial Organization of the Tenascin-C Microenvironment in Experimental and Human Cancer. Cell Adh. Migr..

[244] Sun Z., Velázquez-Quesada I., Murdamoothoo D., Ahowesso C., Yilmaz A., Spenlé C., Averous G., Erne W., Oberndorfer F., Oszwald A. (2019). Tenascin-C Increases Lung Metastasis by Impacting Blood Vessel Invasions. Matrix Biol..

[245] Ming X., Qiu S., Liu X., Li S., Wang Y., Zhu M., Li N., Luo P., Liang C., Tu J. (2019). Prognostic Role of Tenascin-C for Cancer Outcome: A Meta-Analysis. Technol. Cancer Res. Treat..

[246] Yang Z.-T., Yeo S.-Y., Yin Y.-X., Lin Z.-H., Lee H.-M., Xuan Y.-H., Cui Y., Kim S.-H. (2016). Tenascin-C, a Prognostic Determinant of Esophageal Squamous Cell Carcinoma. PLoS ONE.

[247] Qi J., Esfahani D.R., Huang T., Ozark P., Bartom E., Hashizume R., Bonner E.R., An S., Horbinski C.M., James C.D. (2019). Tenascin-C Expression Contributes to Pediatric Brainstem Glioma Tumor Phenotype and Represents a Novel Biomarker of Disease. Acta Neuropathol. Commun..

[248] Kang X., Xu E., Wang X., Qian L., Yang Z., Yu H., Wang C., Ren C., Wang Y., Lu X. (2021). Tenascin-c Knockdown Suppresses Vasculogenic Mimicry of Gastric Cancer by Inhibiting ERK- Triggered EMT. Cell Death Dis..

[249] Ni W.-D., Yang Z.-T., Cui C.-A., Cui Y., Fang L.-Y., Xuan Y.-H. (2017). Tenascin-C Is a Potential Cancer-Associated Fibroblasts Marker and Predicts Poor Prognosis in Prostate Cancer. Biochem. Biophys. Res. Commun..

[250] Yang Z., Ni W., Cui C., Fang L., Xuan Y. (2017). Tenascin C Is a Prognostic Determinant and Potential Cancer-Associated Fibroblasts Marker for Breast Ductal Carcinoma. Exp. Mol. Pathol..

[251] Hashimoto M., Uesugi N., Osakabe M., Yanagawa N., Otsuka K., Kajiwara Y., Ueno H., Sasaki A., Sugai T. (2021). Expression Patterns of Microenvironmental Factors and Tenascin-C at the Invasive Front of Stage II and III Colorectal Cancer: Novel Tumor Prognostic Markers. Front. Oncol..

[252] Li M., Peng F., Li G., Fu Y., Huang Y., Chen Z., Chen Y. (2016). Proteomic Analysis of Stromal Proteins in Different Stages of Colorectal Cancer Establishes Tenascin-C as a Stromal Biomarker for Colorectal Cancer Metastasis. Oncotarget.

[253] Yang Z., Zhang C., Qi W., Cui C., Cui Y., Xuan Y. (2018). Tenascin-C as a Prognostic Determinant of Colorectal Cancer through Induction of Epithelial-to-Mesenchymal Transition and Proliferation. Exp. Mol. Pathol..

[254] Dhaouadi S., Ben Abderrazek R., Loustau T., Abou-Faycal C., Ksouri A., Erne W., Murdamoothoo D., Mörgelin M., Kungl A., Jung A. (2021). Novel Human Tenascin-C Function-Blocking Camel Single Domain Nanobodies. Front. Immunol..

[255] Spenlé C., Loustau T., Murdamoothoo D., Erne W., Beghelli-de la Forest Divonne S., Veber R., Petti L., Bourdely P., Mörgelin M., Brauchle E.-M. (2020). Tenascin-C Orchestrates an Immune-Suppressive Tumor Microenvironment in Oral Squamous Cell Carcinoma. Cancer Immunol. Res..

[256] Tomko L.A., Hill R.C., Barrett A., Szulczewski J.M., Conklin M.W., Eliceiri K.W., Keely P.J., Hansen K.C., Ponik S.M. (2018). Targeted Matrisome Analysis Identifies Thrombospondin-2 and Tenascin-C in Aligned Collagen Stroma from Invasive Breast Carcinoma. Sci. Rep..

[257] Chiquet-Ehrismann R., Kalla P., Pearson C.A. (1989). Participation of Tenascin and Transforming Growth Factor-Beta in Reciprocal Epithelial-Mesenchymal Interactions of MCF7 Cells and Fibroblasts. Cancer Res..

[258] Maschler S., Grunert S., Danielopol A., Beug H., Wirl G. (2004). Enhanced Tenascin-C Expression and Matrix Deposition during Ras/TGF-Beta-Induced Progression of Mammary Tumor Cells. Oncogene.

[259] Katoh D., Nagaharu K., Shimojo N., Hanamura N., Yamashita M., Kozuka Y., Imanaka-Yoshida K., Yoshida T. (2013). Binding of Avβ1 and Avβ6 Integrins to Tenascin-C Induces Epithelial-Mesenchymal Transition-like Change of Breast Cancer Cells. Oncogenesis.

[260] Takahashi Y., Sawada G., Kurashige J., Matsumura T., Uchi R., Ueo H., Ishibashi M., Takano Y., Akiyoshi S., Iwaya T. (2013). Tumor-Derived Tenascin-C Promotes the Epithelial–Mesenchymal Transition in Colorectal Cancer Cells. Anticancer Res..

[261] Cai J., Du S., Wang H., Xin B., Wang J., Shen W., Wei W., Guo Z., Shen X. (2017). Tenascin-C Induces Migration and Invasion through JNK/c-Jun Signalling in Pancreatic Cancer. Oncotarget.

[262] Hawkins A.G., Julian C.M., Konzen S., Treichel S., Lawlor E.R., Bailey K.M. (2019). Microenvironmental Factors Drive Tenascin C and Src Cooperation to Promote Invadopodia Formation in Ewing Sarcoma. Neoplasia.

[263] Qian S., Tan X., Liu X., Liu P., Wu Y. (2019). Exosomal Tenascin-c Induces Proliferation and Invasion of Pancreatic Cancer Cells by WNT Signaling. OncoTargets Ther..

[264] Sarkar S., Mirzaei R., Zemp F.J., Wei W., Senger D.L., Robbins S.M., Yong V.W. (2017). Activation of NOTCH Signaling by Tenascin-C Promotes Growth of Human Brain Tumor-Initiating Cells. Cancer Res..

[265] Yang Z., Zhang C., Feng Y., Qi W., Cui Y., Xuan Y. (2019). Tenascin-C Is Involved in Promotion of Cancer Stemness via the Akt/HIF1α Axis in Esophageal Squamous Cell Carcinoma. Exp. Mol. Pathol..

[266] Katoh D., Kozuka Y., Noro A., Ogawa T., Imanaka-Yoshida K., Yoshida T. (2020). Tenascin-C Induces Phenotypic Changes in Fibroblasts to Myofibroblasts with High Contractility through the Integrin Avβ1/Transforming Growth Factor β/SMAD Signaling Axis in Human Breast Cancer. Am. J. Pathol..

[267] Yeo S.-Y., Lee K.-W., Shin D., An S., Cho K.-H., Kim S.-H. (2018). A Positive Feedback Loop Bi-Stably Activates Fibroblasts. Nat. Commun..

[268] Mirzaei R., Sarkar S., Dzikowski L., Rawji K.S., Khan L., Faissner A., Bose P., Yong V.W. (2018). Brain Tumor-Initiating Cells Export Tenascin-C Associated with Exosomes to Suppress T Cell Activity. Oncoimmunology.

[269] Jachetti E., Caputo S., Mazzoleni S., Brambillasca C.S., Parigi S.M., Grioni M., Piras I.S., Restuccia U., Calcinotto A., Freschi M. (2015). Tenascin-C Protects Cancer Stem-like Cells from Immune Surveillance by Arresting T-Cell Activation. Cancer Res..

[270] Murdamoothoo D., Sun Z., Yilmaz A., Riegel G., Abou-Faycal C., Deligne C., Velazquez-Quesada I., Erne W., Nascimento M., Mörgelin M. (2021). Tenascin-C Immobilizes Infiltrating T Lymphocytes through CXCL12 Promoting Breast Cancer Progression. EMBO Mol. Med..

[271] Guttery D.S., Shaw J.A., Lloyd K., Pringle J.H., Walker R.A. (2010). Expression of Tenascin-C and Its Isoforms in the Breast. Cancer Metastasis. Rev..

[272] Herold-Mende C., Mueller M.M., Bonsanto M.M., Schmitt H.P., Kunze S., Steiner H.-H. (2002). Clinical Impact and Functional Aspects of Tenascin-C Expression during Glioma Progression. Int. J. Cancer.

[273] Berndt A., Anger K., Richter P., Borsi L., Brack S., Silacci M., Franz M., Wunderlich H., Gajda M., Zardi L. (2006). Differential Expression of Tenascin-C Splicing Domains in Urothelial Carcinomas of the Urinary Bladder. J. Cancer Res. Clin. Oncol..

[274] Richter P., Tost M., Franz M., Altendorf-Hofmann A., Junker K., Borsi L., Neri D., Kosmehl H., Wunderlich H., Berndt A. (2009). B and C Domain Containing Tenascin-C: Urinary Markers for Invasiveness of Urothelial Carcinoma of the Urinary Bladder?. J. Cancer Res. Clin. Oncol..

[275] Wilson K.E., Langdon S.P., Lessells A.M., Miller W.R. (1996). Expression of the Extracellular Matrix Protein Tenascin in Malignant and Benign Ovarian Tumours. Br. J. Cancer.

[276] Borsi L., Carnemolla B., Nicolò G., Spina B., Tanara G., Zardi L. (1992). Expression of Different Tenascin Isoforms in Normal, Hyperplastic and Neoplastic Human Breast Tissues. Int. J. Cancer.

[277] Tsunoda T., Inada H., Kalembeyi I., Imanaka-Yoshida K., Sakakibara M., Okada R., Katsuta K., Sakakura T., Majima Y., Yoshida T. (2003). Involvement of Large Tenascin-C Splice Variants in Breast Cancer Progression. Am. J. Pathol..

[278] Hagiwara K., Harimoto N., Yokobori T., Muranushi R., Hoshino K., Gantumur D., Yamanaka T., Ishii N., Tsukagoshi M., Igarashi T. (2020). High Co-Expression of Large Tenascin C Splice Variants in Stromal Tissue and Annexin A2 in Cancer Cell Membranes Is Associated with Poor Prognosis in Pancreatic Cancer. Ann. Surg. Oncol..

[279] Parekh K., Ramachandran S., Cooper J., Bigner D., Patterson A., Mohanakumar T. (2005). Tenascin-C, over Expressed in Lung Cancer down Regulates Effector Functions of Tumor Infiltrating Lymphocytes. Lung Cancer.

[280] Hancox R.A., Allen M.D., Holliday D.L., Edwards D.R., Pennington C.J., Guttery D.S., Shaw J.A., Walker R.A., Pringle J.H., Jones J.L. (2009). Tumour-Associated Tenascin-C Isoforms Promote Breast Cancer Cell Invasion and Growth by Matrix Metalloproteinase-Dependent and Independent Mechanisms. Breast Cancer Res..

[281] Howeedy A.A., Virtanen I., Laitinen L., Gould N.S., Koukoulis G.K., Gould V.E. (1990). Differential Distribution of Tenascin in the Normal, Hyperplastic, and Neoplastic Breast. Lab. Investig..

[282] Sakai T., Kawakatsu H., Hirota N., Yokoyama T., Sakakura T., Saito M. (1993). Specific Expression of Tenascin in Human Colonic Neoplasms. Br. J. Cancer.

[283] Redondo-García S., Peris-Torres C., Caracuel-Peramos R., Rodríguez-Manzaneque J.C. (2021). ADAMTS Proteases and the Tumor Immune Microenvironment: Lessons from Substrates and Pathologies. Matrix Biol. Plus.

[284] Saha N., Robev D., Himanen J.P., Nikolov D.B. (2019). ADAM Proteases: Emerging Role and Targeting of the Non-Catalytic Domains. Cancer Lett..

[285] McKerrow J.H., Bhargava V., Hansell E., Huling S., Kuwahara T., Matley M., Coussens L., Warren R. (2000). A Functional Proteomics Screen of Proteases in Colorectal Carcinoma. Mol. Med..

[286] Bates A.L., Pickup M.W., Hallett M.A., Dozier E.A., Thomas S., Fingleton B. (2015). Stromal Matrix Metalloproteinase 2 Regulates Collagen Expression and Promotes the Outgrowth of Experimental Metastases. J. Pathol..

[287] Catteau X., Simon P., Noël J.-C. (2016). Stromal Expression of Matrix Metalloproteinase 2 in Cancer-Associated Fibroblasts Is Strongly Related to Human Epidermal Growth Factor Receptor 2 Status in Invasive Breast Carcinoma. Mol. Clin. Oncol..

[288] Del Casar J.M., González L.O., Alvarez E., Junquera S., Marín L., González L., Bongera M., Vázquez J., Vizoso F.J. (2009). Comparative Analysis and Clinical Value of the Expression of Metalloproteases and Their Inhibitors by Intratumor Stromal Fibroblasts and Those at the Invasive Front of Breast Carcinomas. Breast Cancer Res. Treat..

[289] Eck S.M., Côté A.L., Winkelman W.D., Brinckerhoff C.E. (2009). CXCR4 and Matrix Metalloproteinase-1 Are Elevated in Breast Carcinoma-Associated Fibroblasts and in Normal Mammary Fibroblasts Exposed to Factors Secreted by Breast Cancer Cells. Mol. Cancer Res..

[290] Panagopoulos V., Leach D.A., Zinonos I., Ponomarev V., Licari G., Liapis V., Ingman W.V., Anderson P., DeNichilo M.O., Evdokiou A. (2017). Inflammatory Peroxidases Promote Breast Cancer Progression in Mice via Regulation of the Tumour Microenvironment. Int. J. Oncol..

[291] Jackson B.C., Nebert D.W., Vasiliou V. (2010). Update of Human and Mouse Matrix Metalloproteinase Families. Hum. Genomics..

[292] Cui N., Hu M., Khalil R.A. (2017). Biochemical and Biological Attributes of Matrix Metalloproteinases. Prog. Mol. Biol. Transl. Sci..

[293] Harper E., Bloch K.J., Gross J. (1971). The Zymogen of Tadpole Collagenase. Biochemistry.

[294] Becker J.W., Marcy A.I., Rokosz L.L., Axel M.G., Burbaum J.J., Fitzgerald P.M., Cameron P.M., Esser C.K., Hagmann W.K., Hermes J.D. (1995). Stromelysin-1: Three-Dimensional Structure of the Inhibited Catalytic Domain and of the C-Truncated Proenzyme. Protein Sci..

[295] Pei D., Weiss S.J. (1996). Transmembrane-Deletion Mutants of the Membrane-Type Matrix Metalloproteinase-1 Process Progelatinase A and Express Intrinsic Matrix-Degrading Activity. J. Biol. Chem..

[296] Van Wart H.E., Birkedal-Hansen H. (1990). The Cysteine Switch: A Principle of Regulation of Metalloproteinase Activity with Potential Applicability to the Entire Matrix Metalloproteinase Gene Family. Proc. Natl. Acad. Sci. USA.

[297] Ferrari R., Martin G., Tagit O., Guichard A., Cambi A., Voituriez R., Vassilopoulos S., Chavrier P. (2019). MT1-MMP Directs Force-Producing Proteolytic Contacts That Drive Tumor Cell Invasion. Nat. Commun..

[298] Franchi M., Piperigkou Z., Karamanos K.-A., Franchi L., Masola V. (2020). Extracellular Matrix-Mediated Breast Cancer Cells Morphological Alterations, Invasiveness, and Microvesicles/Exosomes Release. Cells.

[299] Nakahara H., Howard L., Thompson E.W., Sato H., Seiki M., Yeh Y., Chen W.T. (1997). Transmembrane/Cytoplasmic Domain-Mediated Membrane Type 1-Matrix Metalloprotease Docking to Invadopodia Is Required for Cell Invasion. Proc. Natl. Acad. Sci. USA.

[300] Watanabe A., Hoshino D., Hosino D., Koshikawa N., Seiki M., Suzuki T., Ichikawa K. (2013). Critical Role of Transient Activity of MT1-MMP for ECM Degradation in Invadopodia. PLoS Comput. Biol..

[301] Gould C.M., Courtneidge S.A. (2014). Regulation of Invadopodia by the Tumor Microenvironment. Cell Adh. Migr..

[302] Peláez R., Pariente A., Pérez-Sala Á., Larrayoz I.M. (2019). Integrins: Moonlighting Proteins in Invadosome Formation. Cancers.

[303] Alexander N.R., Branch K.M., Parekh A., Clark E.S., Iwueke I.C., Guelcher S.A., Weaver A.M. (2008). Extracellular Matrix Rigidity Promotes Invadopodia Activity. Curr. Biol..

[304] Jerrell R.J., Parekh A. (2016). Matrix Rigidity Differentially Regulates Invadopodia Activity through ROCK1 and ROCK2. Biomaterials.

[305] Knapinska A.M., Fields G.B. (2019). The Expanding Role of MT1-MMP in Cancer Progression. Pharmaceuticals.

[306] Artym V.V., Zhang Y., Seillier-Moiseiwitsch F., Yamada K.M., Mueller S.C. (2006). Dynamic Interactions of Cortactin and Membrane Type 1 Matrix Metalloproteinase at Invadopodia: Defining the Stages of Invadopodia Formation and Function. Cancer Res..

[307] Yu X., Zech T., McDonald L., Gonzalez E.G., Li A., Macpherson I., Schwarz J.P., Spence H., Futó K., Timpson P. (2012). N-WASP Coordinates the Delivery and F-Actin-Mediated Capture of MT1-MMP at Invasive Pseudopods. J. Cell Biol..

[308] Williams K.C., McNeilly R.E., Coppolino M.G. (2014). SNAP23, Syntaxin4, and Vesicle-Associated Membrane Protein 7 (VAMP7) Mediate Trafficking of Membrane Type 1-Matrix Metalloproteinase (MT1-MMP) during Invadopodium Formation and Tumor Cell Invasion. Mol. Biol. Cell.

[309] Castro-Castro A., Marchesin V., Monteiro P., Lodillinsky C., Rossé C., Chavrier P. (2016). Cellular and Molecular Mechanisms of MT1-MMP-Dependent Cancer Cell Invasion. Annu. Rev. Cell Dev. Biol..

[310] Ohuchi E., Imai K., Fujii Y., Sato H., Seiki M., Okada Y. (1997). Membrane Type 1 Matrix Metalloproteinase Digests Interstitial Collagens and Other Extracellular Matrix Macromolecules. J. Biol. Chem..

[311] d’Ortho M.P., Will H., Atkinson S., Butler G., Messent A., Gavrilovic J., Smith B., Timpl R., Zardi L., Murphy G. (1997). Membrane-Type Matrix Metalloproteinases 1 and 2 Exhibit Broad-Spectrum Proteolytic Capacities Comparable to Many Matrix Metalloproteinases. Eur. J. Biochem..

[312] Strongin A.Y., Collier I., Bannikov G., Marmer B.L., Grant G.A., Goldberg G.I. (1995). Mechanism of Cell Surface Activation of 72-KDa Type IV Collagenase. Isolation of the Activated Form of the Membrane Metalloprotease. J. Biol. Chem..

[313] Tokuraku M., Sato H., Murakami S., Okada Y., Watanabe Y., Seiki M. (1995). Activation of the Precursor of Gelatinase A/72 KDa Type IV Collagenase/MMP-2 in Lung Carcinomas Correlates with the Expression of Membrane-Type Matrix Metalloproteinase (MT-MMP) and with Lymph Node Metastasis. Int. J. Cancer.

[314] Sato H., Takino T. (2010). Coordinate Action of Membrane-Type Matrix Metalloproteinase-1 (MT1-MMP) and MMP-2 Enhances Pericellular Proteolysis and Invasion. Cancer Sci..

[315] Henriet P., Emonard H. (2019). Matrix Metalloproteinase-2: Not (Just) a “Hero” of the Past. Biochimie.

[316] Huang H. (2018). Matrix Metalloproteinase-9 (MMP-9) as a Cancer Biomarker and MMP-9 Biosensors: Recent Advances. Sensors.

[317] Aufderklamm S., Hennenlotter J., Rausch S., Bock C., Erne E., Schwentner C., Stenzl A. (2021). Oncological Validation of Bone Turnover Markers C-Terminal Telopeptide of Type I Collagen (1CTP) and Peptides n-Terminal Propeptide of Type I Procollagen (P1NP) in Patients with Prostate Cancer and Bone Metastases. Transl. Androl. Urol..

[318] Lipton A., Leitzel K., Ali S.M., Polimera H.V., Nagabhairu V., Marks E., Richardson A.E., Krecko L., Ali A., Koestler W. (2018). High Turnover of Extracellular Matrix Reflected by Specific Protein Fragments Measured in Serum Is Associated with Poor Outcomes in Two Metastatic Breast Cancer Cohorts. Int. J. Cancer.

[319] Kehlet S.N., Sanz-Pamplona R., Brix S., Leeming D.J., Karsdal M.A., Moreno V. (2016). Excessive Collagen Turnover Products Are Released during Colorectal Cancer Progression and Elevated in Serum from Metastatic Colorectal Cancer Patients. Sci. Rep..

[320] Maquart F.-X., Pasco S., Ramont L., Hornebeck W., Monboisse J.-C. (2004). An Introduction to Matrikines: Extracellular Matrix-Derived Peptides Which Regulate Cell Activity. Implication in Tumor Invasion. Crit. Rev. Oncol. Hematol..

[321] Maquart F.X., Siméon A., Pasco S., Monboisse J.C. (1999). Regulation of cell activity by the extracellular matrix: The concept of matrikines. J. Soc. Biol..

[322] Davis G.E., Bayless K.J., Davis M.J., Meininger G.A. (2000). Regulation of Tissue Injury Responses by the Exposure of Matricryptic Sites within Extracellular Matrix Molecules. Am. J. Pathol..

[323] Ricard-Blum S., Vallet S.D. (2019). Fragments Generated upon Extracellular Matrix Remodeling: Biological Regulators and Potential Drugs. Matrix Biol..

[324] Han X., Caron J.M., Brooks P.C. (2020). Cryptic Collagen Elements as Signaling Hubs in the Regulation of Tumor Growth and Metastasis. J. Cell Physiol..

[325] Kisling A., Lust R.M., Katwa L.C. (2019). What Is the Role of Peptide Fragments of Collagen I and IV in Health and Disease?. Life Sci..

[326] Heinz A., Jung M.C., Duca L., Sippl W., Taddese S., Ihling C., Rusciani A., Jahreis G., Weiss A.S., Neubert R.H.H. (2010). Degradation of Tropoelastin by Matrix Metalloproteinases—Cleavage Site Specificities and Release of Matrikines. FEBS J..

[327] Wells J.M., Gaggar A., Blalock J.E. (2015). MMP Generated Matrikines. Matrix Biol..

[328] Fujita M., Suzuki H., Fukai F. (2021). Involvement of Integrin-Activating Peptides Derived from Tenascin-C in Colon Cancer Progression. World J. Gastrointest. Oncol..

[329] Fujita M., Sasada M., Iyoda T., Fukai F. (2020). Involvement of Integrin-Activating Peptides Derived from Tenascin-C in Cancer Aggression and New Anticancer Strategy Using the Fibronectin-Derived Integrin-Inactivating Peptide. Molecules.

[330] Joshi R., Goihberg E., Ren W., Pilichowska M., Mathew P. (2017). Proteolytic Fragments of Fibronectin Function as Matrikines Driving the Chemotactic Affinity of Prostate Cancer Cells to Human Bone Marrow Mesenchymal Stromal Cells via the A5β1 Integrin. Cell Adh. Migr..

[331] Pagano M., Reboud-Ravaux M. (2011). Cryptic Activities of Fibronectin Fragments, Particularly Cryptic Proteases. Front. Biosci. (Landmark Ed.).

[332] Adair-Kirk T.L., Atkinson J.J., Broekelmann T.J., Doi M., Tryggvason K., Miner J.H., Mecham R.P., Senior R.M. (2003). A Site on Laminin Alpha 5, AQARSAASKVKVSMKF, Induces Inflammatory Cell Production of Matrix Metalloproteinase-9 and Chemotaxis. J. Immunol..

[333] Papadas A., Arauz G., Cicala A., Wiesner J., Asimakopoulos F. (2020). Versican and Versican-Matrikines in Cancer Progression, Inflammation, and Immunity. J. Histochem. Cytochem..

[334] Brassart-Pasco S., Brézillon S., Brassart B., Ramont L., Oudart J.-B., Monboisse J.C. (2020). Tumor Microenvironment: Extracellular Matrix Alterations Influence Tumor Progression. Front. Oncol..

[335] Aikio M., Alahuhta I., Nurmenniemi S., Suojanen J., Palovuori R., Teppo S., Sorsa T., López-Otín C., Pihlajaniemi T., Salo T. (2012). Arresten, a Collagen-Derived Angiogenesis Inhibitor, Suppresses Invasion of Squamous Cell Carcinoma. PLoS ONE.

[336] Hwang-Bo J., Park J.-H., Bae M.G., Chung I.S. (2016). Recombinant Canstatin Inhibits VEGF-A-Induced Lymphangiogenesis and Metastasis in an Oral Squamous Cell Carcinoma SCC-VII Animal Model. Cancer Med..

[337] Ma Y., Wu T., Zhou H., He G., Li Y., Wang B., Guo Q., Chen B., Li W. (2021). Canstatin Represses Glioma Growth by Inhibiting Formation of VM-like Structures. Transl. Neurosci..

[338] Okada M., Yamawaki H. (2019). A Current Perspective of Canstatin, a Fragment of Type IV Collagen Alpha 2 Chain. J. Pharmacol. Sci..

[339] Li K., Shi M., Qin S. (2018). Current Status and Study Progress of Recombinant Human Endostatin in Cancer Treatment. Oncol. Ther..

[340] Liu X., Nie W., Xie Q., Chen G., Li X., Jia Y., Yin B., Qu X., Li Y., Liang J. (2018). Endostatin Reverses Immunosuppression of the Tumor Microenvironment in Lung Carcinoma. Oncol. Lett..

[341] Walia A., Yang J.F., Huang Y.-H., Rosenblatt M.I., Chang J.-H., Azar D.T. (2015). Endostatin’s Emerging Roles in Angiogenesis, Lymphangiogenesis, Disease, and Clinical Applications. Biochim. Biophys. Acta.

[342] Koskimaki J.E., Karagiannis E.D., Tang B.C., Hammers H., Watkins D.N., Pili R., Popel A.S. (2010). Pentastatin-1, a Collagen IV Derived 20-Mer Peptide, Suppresses Tumor Growth in a Small Cell Lung Cancer Xenograft Model. BMC Cancer.

[343] Li Y., Li J., Woo Y.M., Shen Z., Yao H., Cai Y., Lin M.C.-M., Poon W.S. (2017). Enhanced Expression of Vastatin Inhibits Angiogenesis and Prolongs Survival in Murine Orthotopic Glioblastoma Model. BMC Cancer.

[344] Shen Z., Yao C., Wang Z., Yue L., Fang Z., Yao H., Lin F., Zhao H., Sun Y.-J., Bian X.-W. (2016). Vastatin, an Endogenous Antiangiogenesis Polypeptide That Is Lost in Hepatocellular Carcinoma, Effectively Inhibits Tumor Metastasis. Mol. Ther..

[345] Kim M., Lee C., Seo D.Y., Lee H., Horton J.D., Park J., Scherer P.E. (2020). The Impact of Endotrophin on the Progression of Chronic Liver Disease. Exp. Mol. Med..

[346] Cai M., Onoda K., Takao M., Kyoko I.-Y., Shimpo H., Yoshida T., Yada I. (2002). Degradation of Tenascin-C and Activity of Matrix Metalloproteinase-2 Are Associated with Tumor Recurrence in Early Stage Non-Small Cell Lung Cancer. Clin. Cancer Res..

[347] Mai J., Sameni M., Mikkelsen T., Sloane B.F. (2002). Degradation of Extracellular Matrix Protein Tenascin-C by Cathepsin B: An Interaction Involved in the Progression of Gliomas. Biol. Chem..

[348] Kusagawa H., Onoda K., Namikawa S., Yada I., Okada A., Yoshida T., Sakakura T. (1998). Expression and Degeneration of Tenascin-C in Human Lung Cancers. Br. J. Cancer.

[349] Saito Y., Imazeki H., Miura S., Yoshimura T., Okutsu H., Harada Y., Ohwaki T., Nagao O., Kamiya S., Hayashi R. (2007). A Peptide Derived from Tenascin-C Induces Beta1 Integrin Activation through Syndecan-4. J. Biol. Chem..

[350] Tanaka R., Seki Y., Saito Y., Kamiya S., Fujita M., Okutsu H., Iyoda T., Takai T., Owaki T., Yajima H. (2014). Tenascin-C-Derived Peptide TNIIIA2 Highly Enhances Cell Survival and Platelet-Derived Growth Factor (PDGF)-Dependent Cell Proliferation through Potentiated and Sustained Activation of Integrin A5β1. J. Biol. Chem..

[351] Fujita M., Sasada M., Iyoda T., Nagai R., Kudo C., Yamamoto T., Osada S., Kodama H., Fukai F. (2021). Anoikis Resistance Conferred by Tenascin-C-Derived Peptide TNIIIA2 and Its Disruption by Integrin Inactivation. Biochem. Biophys. Res. Commun..

[352] Fujita M., Yamamoto T., Iyoda T., Fujisawa T., Sasada M., Nagai R., Kudo C., Otsuka K., Kamiya S., Kodama H. (2019). Aggressive Progression in Glioblastoma Cells through Potentiated Activation of Integrin A5β1 by the Tenascin-C-Derived Peptide TNIIIA2. Mol. Cancer Ther..

[353] Fujita M., Yamamoto T., Iyoda T., Fujisawa T., Nagai R., Kudo C., Sasada M., Kodama H., Fukai F. (2019). Autocrine Production of PDGF Stimulated by the Tenascin-C-Derived Peptide TNIIIA2 Induces Hyper-Proliferation in Glioblastoma Cells. Int. J. Mol. Sci..

[354] Iyoda T., Fujita M., Fukai F. (2020). Biologically Active TNIIIA2 Region in Tenascin-C Molecule: A Major Contributor to Elicit Aggressive Malignant Phenotypes From Tumors/Tumor Stroma. Front. Immunol..

[355] Suzuki H., Sasada M., Kamiya S., Ito Y., Watanabe H., Okada Y., Ishibashi K., Iyoda T., Yanaka A., Fukai F. (2017). The Promoting Effect of the Extracellular Matrix Peptide TNIIIA2 Derived from Tenascin-C in Colon Cancer Cell Infiltration. Int. J. Mol. Sci..

[356] Fukai F., Hasebe S., Ueki M., Mutoh M., Ohgi C., Takahashi H., Takeda K., Katayama T. (1997). Identification of the Anti-Adhesive Site Buried within the Heparin-Binding Domain of Fibronectin. J. Biochem..

[357] Shapiro S.D., Endicott S.K., Province M.A., Pierce J.A., Campbell E.J. (1991). Marked Longevity of Human Lung Parenchymal Elastic Fibers Deduced from Prevalence of D-Aspartate and Nuclear Weapons-Related Radiocarbon. J. Clin. Investig..

[358] Heinz A. (2020). Elastases and Elastokines: Elastin Degradation and Its Significance in Health and Disease. Crit. Rev. Biochem. Mol. Biol..

[359] Ntayi C., Labrousse A.-L., Debret R., Birembaut P., Bellon G., Antonicelli F., Hornebeck W., Bernard P. (2004). Elastin-Derived Peptides Upregulate Matrix Metalloproteinase-2-Mediated Melanoma Cell Invasion through Elastin-Binding Protein. J. Investig. Dermatol..

[360] Blood C.H., Sasse J., Brodt P., Zetter B.R. (1988). Identification of a Tumor Cell Receptor for VGVAPG, an Elastin-Derived Chemotactic Peptide. J. Cell Biol..

[361] Da Silva J., Lameiras P., Beljebbar A., Berquand A., Villemin M., Ramont L., Dukic S., Nuzillard J.-M., Molinari M., Gautier M. (2018). Structural Characterization and in Vivo Pro-Tumor Properties of a Highly Conserved Matrikine. Oncotarget.

[362] Brassart B., Randoux A., Hornebeck W., Emonard H. (1998). Regulation of Matrix Metalloproteinase-2 (Gelatinase A, MMP-2), Membrane-Type Matrix Metalloproteinase-1 (MT1-MMP) and Tissue Inhibitor of Metalloproteinases-2 (TIMP-2) Expression by Elastin-Derived Peptides in Human HT-1080 Fibrosarcoma Cell Line. Clin. Exp. Metastasis.

[363] Huet E., Brassart B., Cauchard J.-H., Debelle L., Birembaut P., Wallach J., Emonard H., Polette M., Hornebeck W. (2002). Cumulative Influence of Elastin Peptides and Plasminogen on Matrix Metalloproteinase Activation and Type I Collagen Invasion by HT-1080 Fibrosarcoma Cells. Clin. Exp. Metastasis.

[364] Robinet A., Fahem A., Cauchard J.-H., Huet E., Vincent L., Lorimier S., Antonicelli F., Soria C., Crepin M., Hornebeck W. (2005). Elastin-Derived Peptides Enhance Angiogenesis by Promoting Endothelial Cell Migration and Tubulogenesis through Upregulation of MT1-MMP. J. Cell Sci..

[365] Toupance S., Brassart B., Rabenoelina F., Ghoneim C., Vallar L., Polette M., Debelle L., Birembaut P. (2012). Elastin-Derived Peptides Increase Invasive Capacities of Lung Cancer Cells by Post-Transcriptional Regulation of MMP-2 and UPA. Clin. Exp. Metastasis.

[366] Pocza P., Süli-Vargha H., Darvas Z., Falus A. (2008). Locally Generated VGVAPG and VAPG Elastin-Derived Peptides Amplify Melanoma Invasion via the Galectin-3 Receptor. Int. J. Cancer.

[367] Bretaudeau C., Baud S., Dupont-Deshorgue A., Cousin R., Brassart B., Brassart-Pasco S. (2020). AG-9, an Elastin-Derived Peptide, Increases In Vitro Oral Tongue Carcinoma Cell Invasion, through an Increase in MMP-2 Secretion and MT1-MMP Expression, in a RPSA-Dependent Manner. Biomolecules.

[368] Kim H., Kim M., Im S.-K., Fang S. (2018). Mouse Cre-LoxP System: General Principles to Determine Tissue-Specific Roles of Target Genes. Lab. Anim. Res..

[369] Tammela T., Sage J. (2020). Investigating Tumor Heterogeneity in Mouse Models. Annu. Rev. Cancer Biol..

[370] Mohme M., Maire C.L., Riecken K., Zapf S., Aranyossy T., Westphal M., Lamszus K., Fehse B. (2017). Optical Barcoding for Single-Clone Tracking to Study Tumor Heterogeneity. Mol. Ther..

[371] Hynes R.O., Naba A. (2012). Overview of the Matrisome--an Inventory of Extracellular Matrix Constituents and Functions. Cold Spring Harb. Perspect. Biol..

[372] Krasny L., Huang P.H. (2021). Advances in the Proteomic Profiling of the Matrisome and Adhesome. Expert Rev. Proteom..

[373] Horton E.R. (2021). Functional Bioinformatics Analyses of the Matrisome and Integrin Adhesome. Methods Mol. Biol..

[374] Najafi M., Farhood B., Mortezaee K. (2019). Extracellular Matrix (ECM) Stiffness and Degradation as Cancer Drivers. J. Cell Biochem..

[375] Cruz-Acuña R., Vunjak-Novakovic G., Burdick J.A., Rustgi A.K. (2021). Emerging Technologies Provide Insights on Cancer Extracellular Matrix Biology and Therapeutics. iScience.

[376] Poole J.J.A., Mostaço-Guidolin L.B. (2021). Optical Microscopy and the Extracellular Matrix Structure: A Review. Cells.

[377] Gant K.L., Jambor A.N., Li Z., Rentchler E.C., Weisman P., Li L., Patankar M.S., Campagnola P.J. (2021). Evaluation of Collagen Alterations in Early Precursor Lesions of High Grade Serous Ovarian Cancer by Second Harmonic Generation Microscopy and Mass Spectrometry. Cancers.

[378] Micek H.M., Visetsouk M.R., Masters K.S., Kreeger P.K. (2020). Engineering the Extracellular Matrix to Model the Evolving Tumor Microenvironment. iScience.

[379] Meng F., Meyer C.M., Joung D., Vallera D.A., McAlpine M.C., Panoskaltsis-Mortari A. (2019). 3D Bioprinted In Vitro Metastatic Models via Reconstruction of Tumor Microenvironments. Adv. Mater..

[380] Franchi-Mendes T., Eduardo R., Domenici G., Brito C. (2021). 3D Cancer Models: Depicting Cellular Crosstalk within the Tumour Microenvironment. Cancers.

[381] Hoshiba T. (2019). Decellularized Extracellular Matrix for Cancer Research. Materials.

[382] Gentilin E., D’Angelo E., Agostini M., Astolfi L. (2021). Decellularized Normal and Cancer Tissues as Tools for Cancer Research. Cancer Gene Ther..

